# Protecting Against Postsurgery Oral Cancer Recurrence with an Implantable Hydrogel Vaccine for In Situ Photoimmunotherapy

**DOI:** 10.1002/advs.202309053

**Published:** 2024-10-28

**Authors:** Lan Chen, Qiqi Yin, Handan Zhang, Jie Zhang, Guizhu Yang, Lin Weng, Tao Liu, Chenghui Xu, Pengxin Xue, Jinchao Zhao, Han Zhang, Yanli Yao, Xin Chen, Shuyang Sun

**Affiliations:** ^1^ Department of Oral and Maxillofacial‐Head Neck Oncology Shanghai Ninth People's Hospital Shanghai Jiao Tong University School of Medicine College of Stomatology Shanghai Jiao Tong University Shanghai 200011 China; ^2^ National Center for Stomatology National Clinical Research Center for Oral Diseases Shanghai Key Laboratory of Stomatology Shanghai Research Institute of Stomatology Research Unit of Oral and Maxillofacial Regenerative Medicine Chinese Academy of Medical Sciences Shanghai 200011 China; ^3^ School of Chemical Engineering and Technology Shaanxi Key Laboratory of Energy Chemical Process Intensification, Institute of Polymer Science in Chemical Engineering Xi'an Jiaotong University Xi'an 710049 China

**Keywords:** cancer recurrence, CD47, oral squamous cell carcinoma, photodynamic therapy, photoimmunotherapy, photothermal therapy

## Abstract

Oral squamous cell carcinoma (OSCC) often recurs aggressively and metastasizes despite surgery and adjuvant therapy, driven by postoperative residual cancer cells near the primary tumor site. An implantable in situ vaccine hydrogel was designed to target residual OSCC cells post‐tumor removal. This hydrogel serves as a reservoir for the sustained localized release of δ‐aminolevulinic acid (δ‐ALA), enhancing protoporphyrin IX‐mediated photodynamic therapy (PDT), and a polydopamine‐hyaluronic acid composite for photothermal therapy (PTT). Additionally, immune adjuvants, including anti‐CD47 antibodies (aCD47) and CaCO_3_ nanoparticles, are directly released into the resected tumor bed. This approach induces apoptosis of residual OSCC cells through sequential near‐infrared irradiation, promoting calcium interference therapy (CIT). The hydrogel further stimulates immunogenic cell death (ICD), facilitating the polarization of tumor‐associated macrophages from the M2 to the M1 phenotype. This facilitates phagocytosis, dendritic cell activation, robust antigen presentation, and cytotoxic T lymphocyte‐mediated cytotoxicity. In murine OSCC models, the in situ vaccine effectively prevents local recurrence, inhibits orthotopic OSCC growth and pulmonary metastases, and provides long‐term protective immunity against tumor rechalle nge. These findings support postoperative in situ vaccination with a biocompatible hydrogel implant as a promising strategy to minimize residual tumor burden and reduce recurrence risk after OSCC resection.

## Graphical abstract

An implantable hydrogel‐based in situ vaccine provides localized phototherapy and immunotherapy to eliminate residual OSCC cells within the postsurgical cavity, while inducing robust systemic antitumor immunity through immunogenic cell death. By mimicking a personalized immunotherapy node capable of long‐term tunable drug release, this biomaterial‐mediated vaccine stimulates dendritic cell and cytotoxic T cell responses to suppress OSCC recurrence and distant metastasis.

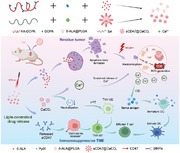

## 1 Introduction

Oral squamous cell carcinoma (OSCC) originates from malignant transformation of epithelial cells lining the oral mucosa.^[^ ^1^ ^]^ Although surgical resection remains the primary treatment for OSCC, complex surrounding anatomy intricately adhered to tumors necessitates careful dissection to preserve organ function.^[^ ^2^ ^]^ However, residual cancer cells frequently persist post‐surgery due to extensive tissue manipulation during resection. The highly flexible nature of oral tissue coupled with infiltrative spread into surrounding structures precludes complete removal. Small residual tumor nests serve as the nidus for recurrence.^[^ ^3^ ^]^ Despite advancements in surgical techniques and adjuvant therapies, residual microtumors persist after surgery, leading to high recurrence rates.^[^ ^2^, ^3^, ^4^ ^]^ Locoregional relapse occurs in ≈50–70% of OSCC cases within 8–12 months postresection.^[^ ^5^ ^]^ The perioperative inflammatory response can additionally activate distant micrometastases before primary tumors become detectable.^[^ ^6^ ^]^ Postsurgical immunosuppression further enables invasion by suppressing immune cell activity.^[^ ^6^, ^7^ ^]^ Recurrent OSCC often acquires increased aggressiveness lacking standard of care.^[^ ^8^ ^]^ To fundamentally restrain relapse, our priority was developing an intervention capable of thoroughly eliminating remaining cancer cells via sustained phototherapy and immunomodulation directly within the high‐risk tumor bed. Residual disease clearance combined with microenvironmental reprogramming is essential to curb OSCC recurrence from its origins.

In addressing this challenge, the direct implantation of therapeutic agents into the tumor cavity emerges as a promising intervention, enhancing the targeting of residual cancer cells postsurgical excision while minimizing widespread systemic exposure.^[^ ^9^ ^]^ Cancer vaccines play a pivotal role in leveraging the host immune system's capabilities to orchestrate potent and targeted anticancer responses.^[^ ^10^ ^]^ Diverse modalities, such as peptides, tumor cells/lysates, tumor‐specific proteins, DNA vaccines, mRNA vaccines, and personalized vaccines coated with tumor cell membranes, have showcased their ability to elicit robust immune reactions against cancer cells.^[^ ^10^, ^11^ ^]^ The presentation of tumor antigens and adjuvants serves as a crucial trigger for initiating anticancer immune responses. To further broaden the understanding of cancer vaccine strategies, recent groundbreaking studies have introduced innovative approaches.^[^ ^12^ ^]^ These studies involved the engineering of bacteria that synergize with programmed death receptor‐1 (PD‐1) antibody, creating a sophisticated interplay that recruits dendritic cells (DCs) and establishes long‐term memory.^[^ ^12^ ^]^ This synergy is accomplished by significantly enhancing the proliferation, activation, and clonal expansion of tumor‐infiltrating lymphocytes and immune memory T cells.^[^ ^12^ ^]^ These findings represent a frontier in cancer immunotherapy, highlighting the dynamic and evolving landscape of strategies aimed at harnessing the immune system against cancer.

The superficial anatomical location of oral squamous cell carcinoma renders tumors readily accessible for localized therapeutic implantation after surgical resection. While chemotherapy or molecularly‐targeted agent loaded implants alone confer limited antitumor efficacy,^[^ ^13^ ^]^ integrating immunotherapies that stimulate immunogenic cell death (ICD) can enhance local cytotoxicity while engaging durable systemic immune responses against residual malignant cells.^[^ ^14^ ^]^ Both sprayable and injectable hydrogels releasing immunomodulators have demonstrated safety and potential for promoting innate and adaptive antitumor immunity after resection, suppressing local recurrence, and impeding metastatic progression.^[^ ^15^ ^]^ Furthermore, implantation of engineered chimeric antigen receptor (CAR) T cells allows continuous manufacture and release of CAR‐T cells directly into high‐risk tumor beds, ensuring on‐site activity against remaining cancer cells while mitigating systemic toxicity.^[^ ^16^ ^]^ These emerging postoperative vaccination approaches highlight the promise of sustained in situ immunomodulation within accessible tumor microenvironments for effectively eradicating residual disease, forestalling relapse, and improving outcomes after cancer surgery.

Capitalizing on the superficial location and frequent residual disease in OSCC postresection,^[^ ^17^ ^]^ we have envisioned an implantable system concurrently tackling multiple therapeutic challenges to suppress recurrence. Strategic integration of photodynamic therapy (PDT), photothermal therapy (PTT), and calcium overload therapy rapidly induces residual cancer cell death. Local delivery of biocompatible CaCO_3_ nanoparticles further neutralizes acidic tumor pH while polarizing immunosuppressive macrophages toward phagocytic M1‐phenotypes. Recognizing OSCC cells evade macrophages by upregulating “don't eat me” signal CD47, the system releases anti‐CD47 antibodies (aCD47) to enhance immune recognition and clearance of malignant cells. Synergistic PDT and PTT stimulate ICD, locally disseminating tumor antigens to activate durable antitumor immunity, thereby forestalling relapse. This multifaceted approach represents a focused strategy combatting complex, multifactorial mechanisms enabling OSCC recurrence within the immunosuppressive tumor microenvironment.

In this work, we engineered an in situ vaccine hydrogel (APHP‐CCCA) protecting against postoperative OSCC recurrence. This biodegradable nanocomposite comprises a photothermal polydopamine‐hyaluronic acid matrix and photosensitizer‐loaded nanoparticles, plus an immunomodulatory calcium alginate hydrogel with aCD47‐tagged CaCO_3_ nanoparticles. Crosslinking enables formation of an integrated system for localized sequential phototherapy to eradicate residual disease. Released aCD47 antibodies stimulate macrophage phagocytosis while synergistic PDT and PTT remodel the microenvironment, eliciting systemic antitumor immunity. In OSCC models, APHP‐CCCA suppressed recurrence and metastatic progression while conferring immunologic memory. This study validates in situ vaccination with multifaceted biomaterials as a promising postoperative strategy coordinating immunogenic cytotoxicity to prevent relapse.

## 2 Results

### 2.1 Synthesis and Characterization of the Nanocomposite In Situ Vaccine

**Figure 1:**
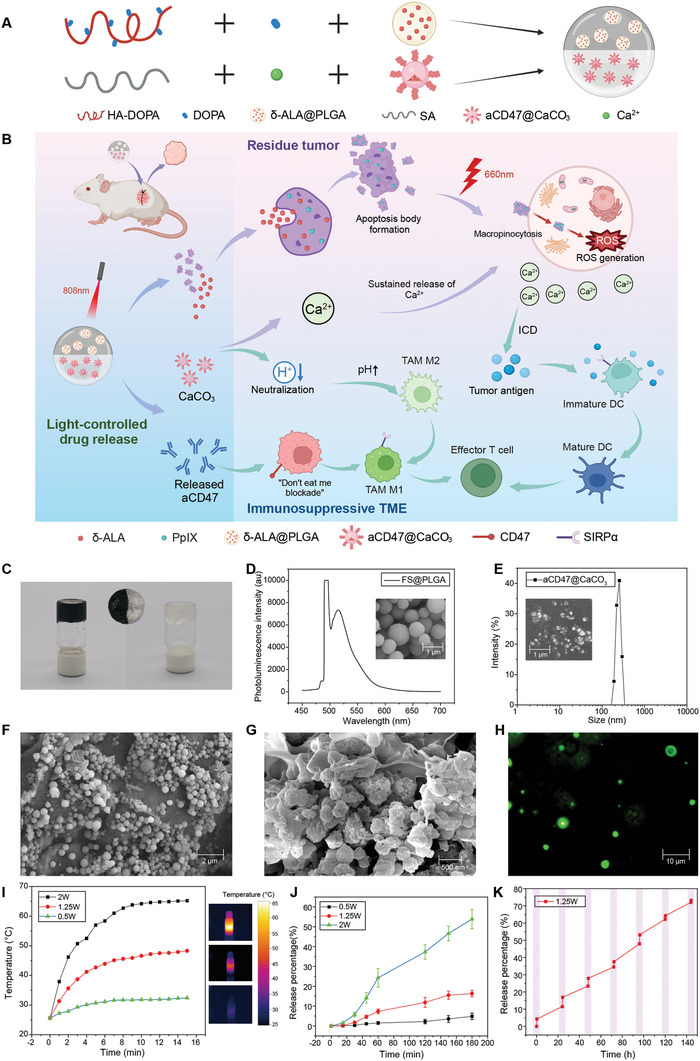
Synthesis and characterization of the implantable in situ vaccine hydrogel. A) Schematic illustration of the construction of APHP‐CCCA nanocomposite hydrogel. B) A schematic illustrating the APHP‐CCCA in situ implantable hydrogel vaccine is presented. Comprising a photothermal polydopamine‐hyaluronic acid matrix, photosensitizer‐loaded PLGA nanoparticles, immunomodulatory aCD47, and CaCO_3_ nanoparticles, the vaccine facilitates PTT, PDT, and calcium overload‐induced ICD. This multifunctional vaccine hydrogel ensures spatiotemporally controlled delivery of synergistic phototherapy, calcium interference, and immunotherapy post‐tumor resection. Its capabilities extend to the elimination of residual cancer cells through apoptotic cytotoxic modalities, concurrently reversing local immunosuppression to stimulate systemic antitumor immunity against recurrence. C) Photographs of APHP‐CCCA nanocomposite hydrogel. D) SEM images and fluorescence spectra of FS@PLGA nanoparticles. Scale bar, 1 µm. E) SEM images and dynamic light scattering particle size distribution of aCD47@CaCO_3_ nanoparticles. Scale bar, 1 µm. F) SEM image of local magnification of APHP photothermal‐photodynamic hydrogel and internal δ‐ALA@PLGA microspheres. Scale bar, 2 µm. G) Locally enlarged SEM image of CCCA immunomodulatory hydrogel and its internal aCD47@CaCO_3_ nanoparticles. Scale bar, 500 nm. H) Fluorescence microscopic image of FITC‐labeled aCD47@CaCO_3_ nanoparticles from immunomodulatory CCCA hydrogel. Scale bar, 10 µm. I) Temperature elevation curves of APHP‐CCCA hydrogel when exposed to 808 nm laser irradiation at varying power densities. J) The time‐dependent release kinetics of δ‐ALA from APHP‐CCCA hydrogel under 808 nm laser irradiation at varying power densities for 180 min. K) The cumulative δ‐ALA release profile of APHP‐CCCA hydrogel irradiated by 808 nm laser at 1.25 W for 15 min daily over 1 week. Data are presented as the mean ± SD; *n* = 3 independent experiments.

The in situ vaccine hydrogel APHP‐CCCA consists of two synergistic components integrated into a unified system. One component (APHP) is a polydopamine‐hyaluronic acid hydrogel loaded with δ‐aminolevulinic acid (δ‐ALA) encapsulated PLGA nanoparticles (δ‐ALA@PLGA), providing photothermal and photodynamic therapy. The other component (CCCA) is a CA hydrogel containing aCD47 conjugated CaCO_3_ nanoparticles (aCD47@CaCO_3_), enabling immunomodulation. Interpenetrating network formation through calcium crosslinking of alginate and catechol‐mediated adhesion of PDA integrates APHP and CCCA into the full APHP‐CCCA hydrogel. This multifunctional integration enables spatiotemporal control over phototherapy and immunotherapy for coordinated cytotoxic effects and immune stimulation (**Figure** **1**A,B). The stepwise fabrication process and integrated structure are depicted (Figure 1A,C). The rational design of APHP‐CCCA synthesizes distinct hydrogel composites into a unified in situ vaccine system for enhanced anticancer functionality.

Amidation created dopamine‐modified hyaluronic acid (HA‐DOPA), confirmed by nuclear magnetic resonance spectroscopy showing characteristic benzene peaks (Figure S1A, Supporting Information). In order to study the morphology and assess the practicality of drug loading of δ‐ALA@PLGA nanoparticles by scanning electron microscopy and fluorescence spectra, compound fluorescein sodium (FS) was used as a substitute for δ‐ALA (Figure 1D). Results indicated that FS@PLGA nanoparticles mainly exhibited uniform 500 nm spheres, while also implied the feasibility of loading δ‐ALA on PLGA, which was evidenced by characteristic fluorescence peaks at 520 nm. Similarly, SEM and dynamic light scattering showed that the size of aCD47@CaCO₃ nanoparticles were approximately 200 nm, confirming its successful synthesis (Figure 1E). The composite gel architecture comprised polymerized dopamine‐modified hyaluronic acid and calcium‐crosslinked alginate (Figure 1C). Drug‐loaded particles were incorporated during gelation. Solutions pregelation appeared transparent (Figure S1B, Supporting Information). Polymerization of HA‐DOPA and CaCl_2_ addition to alginate induced rapid gelation. SEM analyzed morphology and drug loading. APHP gel displayed 10–20 µm pores with adequate PLGA microspheres loaded (Figure 1F; Figure S1C, Supporting Information). Calcium alginate gel exhibited lamellar structure with incorporated CaCO_3_ nanoparticles (Figure 1G; Figure S1D, Supporting Information), confirming efficacious drug‐loaded gel synthesis. Subsequently, its fluorescence microscopy revealed green fluorescence localized to spherical nanoparticles, verifying the successful loading of fluorescein isothiocyanate (FITC)‐labeled aCD47 onto the CaCO_3_ nanoparticles from CCCA hydrogel, consequently proving the successful synthesis of aCD47@CaCO_3_ (Figure 1H).

We then utilized infrared thermal imaging to quantify temperature variations in the APHP‐CCCA hydrogel. We observed temperature elevations of ≈5, 20, and 40 °C at 0.5, 1.25, and 2 W power after 15 min, respectively (Figure 1I). These findings provided evidence of favorable photothermal properties of the hydrogel. Concurrently, we compared drug release from the hydrogel under varying power levels. We observed a significant correlation between exposure duration, applied power, and δ‐ALA release (Figure 1J). The final δ‐ALA release rate from APHP‐CCCA was 52% at 2 W, 14% at 1.25 W, and 4% at 0.5 W during 180 min (Figure 1J). Although 2 W power optimized the photothermal effect and drug release, the 40 °C temperature rise may adversely impact the body normal tissues. Therefore, 1.25 W power is considered the most suitable for the APHP‐CCCA hydrogel. The APHP‐CCCA then underwent controlled release testing at 1.25 W. 1 mg gel in 1 mL pH 6.8 PBS was exposed to an 808 nm laser at 1.25 W. Over 1 week, 15‐min exposures were followed by 24‐h standing periods (Figure 1K). We collected the supernatant before and after each exposure, then utilized fluorescence spectrophotometry to measure δ‐ALA release, which revealed ≈72% cumulative release after the extended controlled release at 1.25 W (Figure 1K; Figure S1E, Supporting Information), indicating this photothermal gel's potential for successful long‐term in vivo treatment.

Calcium carbonate dissolution increases pH by reducing H^+^ ion concentration. To evaluate the pH‐regulating efficacy of APHP‐CCCA hydrogel, we added hydrogel to PBS at pH 6.8 and measured supernatant pH value using a pH meter. Results showed the pH exceeded 7.4 within 1 h and remained elevated over 120 h (Figure S1F, Supporting Information), indicating the hydrogel has favorable sustained pH‐regulating capacity that may facilitate M1 macrophage activation. The encapsulated CD47 antibody was released through gradual dissolution of the CaCO_3_ nanoparticles. FITC‐labeled antibody enabled observation of release using fluorescence photometry. APHP‐CCCA hydrogel in pH 6.8 PBS had supernatant collected over 2 weeks. Fluorescence spectrophotometry determined antibody release rates. Results showed ≈73% cumulative release (Figure S1G,H, Supporting Information), indicating the CaCO_3_ nanoparticles effectively controlled slow release and prolonged antibody retention. These results not only indicated the successful formation of APHP‐CCCA, but also demonstrated their functions of photothermal effect, pH manipulation and aCD47 controlled release for the further photoimmunotherapy of OSCC.

### 2.2 Phototherapeutic Effects and Light‐Controlled Drug Release of APHP‐CCCA

**Figure 2:**
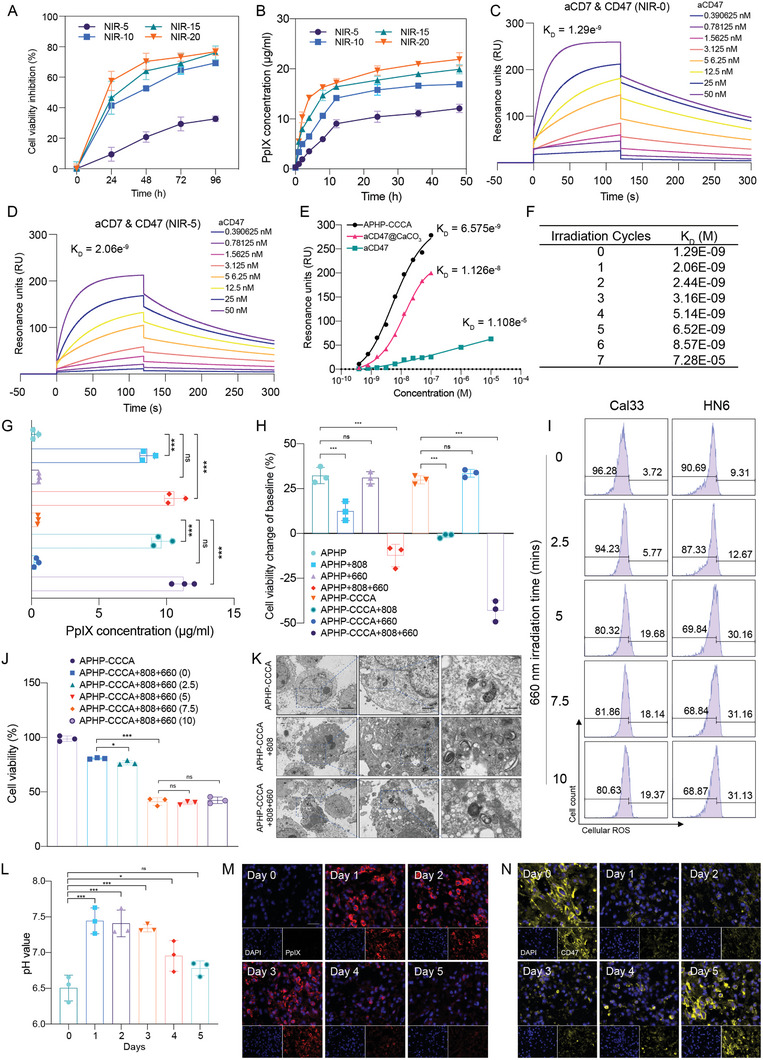
Phototherapeutic effects and light‐controlled drug release of APHP‐CCCA. A) Time‐dependent inhibition of Cal33 cell viability by photothermal therapy using 100 µg mL^−1^ APHP‐CCCA upon 808 nm laser irradiation at 1.25 W cm^−2^ for different durations (5, 10, 15, and 20 min; NIR‐5, NIR‐10, NIR‐15, and NIR‐20). B) Quantitative analysis of intracellular PpIX concentration in Cal33 cells treated with 100 µg mL^−1^ APHP‐CCCA after different durations of 808 nm laser irradiation. C) Dose‐response curve from SPR measurements showing binding of aCD47 released from APHP‐CCCA without laser irradiation to immobilized CD47 protein. D) Dose‐response curve from SPR measurements showing binding of aCD47 released from APHP‐CCCA upon 808 nm laser irradiation at 1.25 W cm^−2^ for 5 min to immobilized CD47 protein. E) SPR sensorgrams showing binding of free aCD47, aCD47 released from aCD47@CaCO_3_ nanoparticles or APHP‐CCCA hydrogel after 808 nm laser irradiation for 5 min to immobilized CD47 protein. F) Dissociation equilibrium constant (K_D_) values of aCD47 released from APHP‐CCCA upon 808 nm laser irradiation at 1.25 W cm^−2^ for different durations determined by SPR analysis. G) Quantitative analysis of intracellular PpIX concentration in Cal33 cells treated with APHP or APHP‐CCCA and irradiated with 808 nm laser at 1.25 W cm^−2^ for 5 min and/or 660 nm laser at 40 mW cm^−2^ for 20 min followed by 24 h culture. H) Viability of Cal33 cells treated with APHP or APHP‐CCCA and irradiated with 808 nm laser at 1.25 W cm^−2^ for 5 min and/or 660 nm laser at 40 mW cm^−2^ for 20 min followed by 24 h culture. I) ROS levels in Cal33 and HN6 cells determined by flow cytometry after treatment with 660 nm laser at 40 mW cm^−2^ for different durations (0, 2.5, 5, 7.5, and 10 min). J) Viability of Cal33 cells treated with APHP‐CCCA and irradiated with 808 nm laser at 1.25 W cm^−2^ for 5 min, followed by irradiation with 660 nm laser at 40 mW cm^−2^ for different durations (0, 2.5, 5, 7.5, and 10 min). K) Representative TEM images of the ultrastructure of Cal33 cells treated with APHP‐CCCA with no NIR irradiation, 5 min of 808 nm laser exposure, or 5 min of 808 nm plus 5 min of 660 nm laser irradiation. Scale bar, 2 µm, 500 nm, and 100 nm. L) Intratumoral pH values on each day for 5 d following surgery. M) Representative fluorescence images of intracellular PpIX in residual tumors on each day for 5 d following surgery after 5 min of 808 nm plus 5 min of 660 nm laser irradiation. Red, PpIX; blue, DAPI. Scale bar, 20 µm. N) Representative fluorescence images of CD47 expression in residual tumor tissue on each day for 5 d following surgery after 5 min of 808 nm plus 5 min of 660 nm laser irradiation. Yellow, CD47; blue, DAPI. Scale bar, 20 µm. Data are presented as the mean ± SD; *n* = 3 independent experiments. *p* values were determined by two‐way ANOVA, Tukey's multiple‐comparison test (ns, not significant; ^*^ *p* < 0.05; ^**^ *p* < 0.01; ^***^ *p* < 0.001).

We first investigated the photothermal effects of the hydrogel. Coincubation of OSCC cells (Cal33 and HN6) with varying concentrations of APHP‐CCCA for 72 h without near‐infrared (NIR) light irradiation showed no cytotoxicity on cell viability (Figure S2A,B, Supporting Information). In contrast, photothermal treatment with APHP‐CCCA induced significant time‐dependent cancer cell death when exposed to an 808 nm laser (1.25 W cm^−2^) for 5, 10, 15, and 20 min (**Figure** **2**A; Figure S2C, Supporting Information). Live/dead staining of the treated cells further verified the PTT efficacy of APHP‐CCCA (Figure S2D,E, Supporting Information). Laser irradiation not only elicited PTT but also triggered light‐controlled drug release from APHP‐CCCA. Under 808 nm laser irradiation (1.25 W cm^−2^), the release of δ‐ALA was significantly correlated with irradiation time, as evidenced by increased intracellular accumulation of the endogenous photosensitizer protoporphyrin IX (PpIX) inside OSCC cells (Figure 2B; Figure S2F, Supporting Information). It was also found that CaCO_3_ nanoparticles were released upon 808 nm laser irradiation, as the pH of OSCC cell supernatant increased and remained alkaline (Figure S2G, Supporting Information). With degradation of CaCO_3_ nanoparticles, FITC‐labeled aCD47 was also released in the OSCC cell supernatant (Figure S2H, Supporting Information).

We also found that after coculturing OSCC cells with APHP or APHP‐CCCA and irradiating with only 660 nm laser for 20 min followed by 24 h of culture, neither PpIX fluorescence nor cell viability were significantly affected (Figure 2G,H). Additionally, we incubated APHP‐CCCA with OSCC cells for 72 h without NIR irradiation and did not observe a significant increase in pH value of the supernatants or release of FITC‐labeled aCD47 (Figure S3D,E, Supporting Information). Based on these results, it appears that light‐controlled release via PTT using an 808 nm laser is responsible for drug release from APHP‐CCCA.

Next, the appropriate laser energy density to apply during PDT was also determined. Cal33 and HN6 cells were treated with APHP‐CCCA and exposed to 808 nm laser irradiation at 1.25 W cm^−2^ for 5 min, then irradiated with a power density of 40 mW cm^−2^ at 660 nm for different times (0, 2.5, 5, 7.5, and 10 min). Intracellular reactive oxygen species (ROS) production was then determined. The light‐irradiated cells (5 min 660 nm NIR irradiation) showed a significant shift in green fluorescence signal, and flow cytometry demonstrated that the mean fluorescence intensity increased 5.29 and 3.24 times compared to unirradiated Cal33 and HN6 cells, suggesting that PDT led to robust ROS production (Figure 2I). Fluorescence imaging further validated ROS generation in OSCC cells under photoirradiation (Figure S3F, Supporting Information). After 5 min of 660 nm laser irradiation, the viability of Cal33 decreased to 53.6% compared to the 660 nm unirradiated group, and the inhibitory effect did not increase significantly with increasing irradiation time (Figure 2J). Transmission electron microscopy (TEM) was then used to examine the ultrastructure of OSCC cells following NIR irradiation. APHP‐CCCA co‐incubated Cal33 cells showed typical apoptotic morphological changes after 5 min of 808 nm followed by 5 min of 660 nm laser irradiation (Figure 2K). Specifically, perinuclear chromatin was compacted into crescents, apoptotic bodies appeared at the cell edges, and cells disintegrated into 300–700 nm vesicles (Figure 2K). Therefore, a laser radiation density of 12 J cm^−2^ (40 mW cm^−2^ for 5 min) is appropriate for PDT in this study.

### 2.3 Optimizing Mild PTT to Preserve aCD47 Activity

Given the potential for elevated temperatures during PTT to deactivate aCD47,^[^ ^18^ ^]^ we intentionally tailored our approach to utilize mild PTT combined with short‐term, repeated laser irradiation cycles.

The selection of 1.25 W cm^−2^ power was a balanced consideration between the thermal effects and drug release outcomes. While 2 W cm^−2^ power demonstrated optimal photothermal effects and drug release, the resultant temperature increase of up to 40 °C raised concerns about adverse effects on normal tissue and potential inactivation of aCD47 (Figure 1I). Thus, we consciously avoided choosing 2 W cm^−2^ due to its likelihood of inducing rapid and intense temperature elevation, leading to aCD47 inactivation. Similarly, we opted against 0.5 W cm^−2^ due to inadequate photothermal effects, as prolonged irradiation exceeding 12 h failed to raise temperatures above 35 °C (Figure 1I), coupled with notably low δ‐ALA release efficiency from APHP‐CCCA (Figure 1J). Regarding the release of CaCO_3_ and PpIX, we observed that irradiation at 1.25 W cm^−2^ for daily sessions over one week resulted in ≈80% cumulative δ‐ALA release profile from APHP‐CCCA hydrogel (Figure 1k). Consequently, we selected 1.25 W cm^−2^ power as it offered a favorable compromise.

Through meticulous selection of irradiation time and cycles, we achieved mild PTT effects to promote immune activation without compromising aCD47 activity, while ensuring high local drug release. We systematically evaluated the impact of different 808 nm laser irradiation exposure times and cycles on aCD47 activity using surface plasmon resonance (SPR) analysis, enabling sensitive and quantitative measurements of antibody–antigen binding affinity (Figure 2C,D; Figure S3A–C, Supporting Information). Our findings revealed that 5 min of laser irradiation did not significantly alter aCD47 binding affinity for immobilized CD47 protein (Figure 2C,D). However, prolonged irradiation exceeding 10 min substantially reduced binding affinity, indicating that longer single exposures above 5 min could negatively affect aCD47 function (Figure S3A–C, Supporting Information). We further determined that up to 6 cycles of repeated 5 min irradiation preserved antibody activity before a significant decline in affinity was observed (Figure 2F), demonstrating the feasibility of employing multiple short exposures while maintaining aCD47 efficacy. Furthermore, we compared the released aCD47 from CaCO_3_ nanoparticles and APHP‐CCCA hydrogels after 5 min PTT exposures (Figure 2E). Over 24 h, the released aCD47 exhibited greater stability compared to free aCD47 incubated under the same conditions, supporting our light‐triggered release approach from CaCO_3_ nanoparticles and CaCO_3_ degradation in the acidic tumor microenvironment as contributors to sustained aCD47 efficacy (Figure 2E). We also found that 660 nm laser irradiation, at different powers and durations, does not affect the activity of aCD47 (Figure S3G, Supporting Information).

In summary, the meticulous optimization of mild PTT parameters facilitates controlled release kinetics and preservation of potent aCD47 immunotherapeutic function, enhancing the efficacy of our therapeutic approach.

### 2.4 Multiple‐Cycle Laser Irradiation Enables Long‐Term APHP‐CCCA Tumor Retention

We then examined the interval time and irradiation cycles of PTT/PDT after the in situ vaccination of APHP‐CCCA to enable long‐term tumor retention of various agents. To mimic incomplete tumor removal in the clinic,^[^ ^19^ ^]^ SCC7 cells were inoculated into the flanks of mice, and ≈90% of the tumor was surgically resected when the tumor volume reached ≈300 mm^3^. After surgical resection, APHP‐CCCA was implanted in situ, followed by laser irradiation (808 nm for 5 min, 1.25 W cm^−2^, and 660 nm for 5 min, 40 mW cm^−2^). In vivo drug release was monitored for the next 5 d.

As CaCO_3_ nanoparticles have been shown to effectively modulate tumor acidity,^[^ ^20^ ^]^ we used a pH microelectrode to measure changes in pH values inside tumors for 5 d following surgery to assess CaCO_3_ nanoparticle retention. We found that the tumor pH value on the first day postsurgery was significantly higher than before implantation, remained stable on days 2–3, and began to decline on day 4 (Figure 2L). Fluorescence imaging of residual tumor tissue revealed that PpIX levels increased significantly after laser irradiation and remained elevated until 3 d postsurgery (Figure 2M; Figure S4A, Supporting Information). Similarly, CD47 activity increased slowly after laser irradiation and decreased significantly on day 4 postsurgery, as determined by immunofluorescence staining of CD47 in residual tumor tissues (Figure 2N; Figure S4B, Supporting Information). Based on these results, the appropriate time interval between two laser irradiations is 3 d to maintain high drug levels in the tumor cavity.

We then examined the appropriate number of irradiation cycles to enable long‐term tumor retention of agents. We implanted APHP‐CCCA in situ in mice with incompletely resected SCC7 tumors, followed by multiple cycles of laser irradiation (808 nm for 5 min, 1.25 W cm^−2^, and 660 nm for 5 min, 40 mW cm^−2^) every 3 d. The second day after each irradiation, we measured PpIX levels to assess δ‐ALA release, pH levels within the tumor to assess CaCO_3_ nanoparticle release, and CD47 expression to assess aCD47 release (Figure S4C–E, Supporting Information). After the 7th laser irradiation, we found no significant changes in tumor pH, indicating no new CaCO_3_ nanoparticle release (Figure S4C, Supporting Information). Minimal PpIX fluorescence revealed only small δ‐ALA retention in the tumor (Figure S4D, Supporting Information). Increased CD47 expression also indicates completion of aCD47 release (Figure S4E, Supporting Information). Thus, six laser irradiation cycles will ensure light‐triggered release from the in situ vaccine, enabling long‐term retention of agents within the postoperative cavity.

### 2.5 In Vivo Antitumor Efficacy of APHP‐CCCA Hydrogel

**Figure 3:**
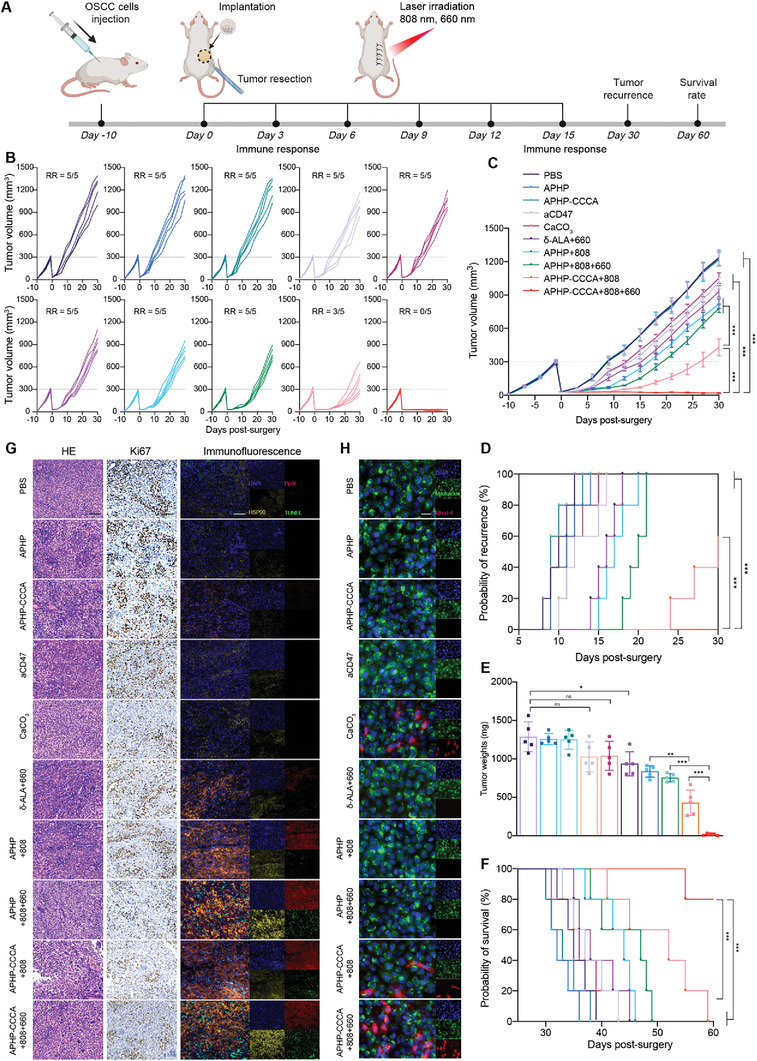
In vivo tumor recurrence suppression efficacy of APHP‐CCCA hydrogel implantation postsurgery.A) Schematic illustration of the animal experimental design. NIR irradiation: 808 nm laser at 1.25 W cm^−2^ for 5 min and/or 660 nm laser at 40 mW cm^−2^ for 5 min. B) Individual tumor growth curves of mice in different groups over 30 d following surgery. RR: recurrence rate. C) Average tumor growth curves of different groups during 30 d after surgery. Data are shown as mean ± SEM (*n* = 5 mice per group). D) Percentage of mice with tumor recurrence in each group at 30 d after surgery (*n* = 5 mice per group). E) Tumor weights of recurrent tumors on day 30 after surgery. Data are shown as mean ± SD (*n* = 5 mice per group). F) Long‐term survival percentages of mice in each group over 60 d following surgery (*n* = 5 mice per group). G) H&E staining of residual tumors in each group at 30 d after surgery; scale bar, 200 µm. Ki67 immunohistochemical staining of residual tumors in each group at third day after surgery; scale bar, 200 µm. Representative fluorescence images showing intracellular PpIX, CD47 expression, HSP90 expression and TUNEL in residual tumor tissue from each group at 30 d after surgery; scale bar, 200 µm. Blue: DAPI; red: PpIX; yellow: HSP90; green: TUNEL. H) Representative fluorescence images showing calcium influx and mitochondrial staining in live Cal33 cells from each group. Scale bar, 20 µm. Blue: DAPI; red: Rhod‐4 AM stain indicates calcium influx; green: MitoTracker Green FM stain indicates mitochondrial localization and membrane potential. Data are presented as the mean ± SD; *n* ≥ 3 independent experiments. *p* values were determined by two‐way ANOVA, Tukey's multiple‐comparison test (ns, not significant; ^*^ *p* < 0.05; ^**^ *p* < 0.01; ^***^ *p* < 0.001).

To evaluate the antitumor efficacy of APHP‐CCCA hydrogel, we utilized an incompletely resected SCC7‐tumor mouse model^[^ ^21^ ^]^ and examined suppression of tumor recurrence postsurgery (**Figure** **3**A). To mimic incomplete tumor resection occurring clinically, SCC7 cells were injected into the right flank of mice, and ≈90% of the tumor was surgically resected when tumor volume reached 300 mm^3^. This was followed by implanting hydrogel at the surgical resection site before suturing (Figure 3A). Mice were randomly divided into the following 10 treatment groups: PBS, aCD47, CaCO_3_, δ‐ALA with 660 nm NIR (δ‐ALA+660), APHP hydrogel with no NIR, 808 nm NIR, or 808 and 660 nm NIR (APHP, APHP+808, APHP+808+660), APHP‐CCCA hydrogel with no NIR, 808 nm NIR, or 808 and 660 nm NIR (APHP‐CCCA, APHP‐CCCA+808, APHP‐CCCA+808+660). Laser irradiation was performed on days 0, 3, 6, 9, 12, and 15 postsurgery. Tumor volume and body weight were measured every three days, and two‐month survival rate was assessed. Upon reaching a humane endpoint or the study endpoint, mice were euthanized.

The “δ‐ALA+660”, “APHP+808”, and “APHP+808+660” groups all received 6 rounds of laser irradiation, and tumor volumes increased significantly on days 9, 12, and 15 after surgery, respectively (Figure 3B–E). Compared to the PBS group, the tumor growth inhibition rates of the three groups were 24.60%, 33.57%, and 36.90% (Figure 3B–E). Among the three groups, the “δ‐ALA+660” group had the lowest antitumor effect, likely due to rapid diffusion and metabolism of free δ‐ALA within the tumor cavity, preventing continuous PDT effect and antitumor activity (Figure 3B–E). In the “APHP+808+660” group, tumor recurrence was partially suppressed, suggesting continuous δ‐ALA release via hydrogel and multiple PTT/PDT rounds are necessary for antitumor activity (Figure 3B–E). The “APHP‐CCCA+808” group had significantly reduced tumor recurrence compared to PBS, but 3 of 5 mice still had recurrence by day 30, suggesting lack of long‐term antitumor effects (Figure 3B–E). Compared to the other 9 groups, none of the “APHP‐CCCA+808+660” mice had tumor recurrence, with 2 of 5 mice having almost no tumor and 81.86% tumor regression (Figure 3B–E). This group had the highest long‐term survival, with 100% survival at day 50 and 80% at day 60 (Figure 3F).

Residual tumor cells are a major factor in tumor recurrence. We used in situ vaccination to kill residual tumor cells via PTT, PDT, and CIT. When tumor cells undergo PTT‐induced hyperthermia, they produce high levels of heat shock protein 90 (HSP90), which protects against heat damage.^[^ ^22^ ^]^ HSP90 expression was assessed in tumor tissues from each group on day 3 postsurgery using immunofluorescence staining (Figure 3G). HSP90 expression in the “APHP+808”, “APHP+808+660”, “APHP‐CCCA+808”,″ and “APHP‐CCCA+808+660” groups exceeded PBS by 2.16, 2.89, 2.42, and 3.33 times, respectively, indicating 808 nm NIR could induce PTT‐induced cell death (Figure 3G; Figure S5A, Supporting Information). Thermal imaging of SCC7 tumor‐bearing mice following 5 min of 808 nm laser irradiation after treatment with PBS, APHP, or APHP‐CCCA hydrogels corroborated a gradual increase in temperature at the tumor site in mice treated with APHP or APHP‐CCCA combined with 808 nm laser. The temperature plateaued around 44 °C after 5 min of laser exposure, validating the mild photothermal therapy approach employed in this study (Figure S5C,D, Supporting Information). Compared to PBS, the “APHP+808+660” and “APHP‐CCCA+808+660” groups showed significant TUNEL‐positive cells (Figure 3G; Figure S5B, Supporting Information) and increased PpIX production (Figure 3G; Figure S5E, Supporting Information), suggesting in situ vaccination exerts antitumor effects via PDT‐induced cell death. The in situ hydrogel decomposed CaCO_3_ nanoparticles over time to produce high Ca^2+^ levels, promoting local PTT heating so Ca^2+^ enters and accumulates in mitochondria, causing dysfunction. The “CaCO_3_”, “APHP‐CCCA+808”, and “APHP‐CCCA+808+660” groups showed obvious mitochondrial dysfunction (upregulated cytochrome c and downregulated Bcl‐2) (Figure S5F,G, Supporting Information). Moreover, in vitro experiments with live OSCC cells showed “APHP‐CCCA+808+660” markedly increased cellular calcium influx versus PBS (Figure 3H; Figure S5H, Supporting Information). These findings indicate postoperative in situ hydrogel implantation can achieve sustained drug retention in the tumor cavity via NIR‐triggered release, thereby suppressing OSCC recurrence.

### 2.6 Immunomodulatory Effects of APHP‐CCCA in the Low‐Immunogenic OSCC Model

The immunosuppressive microenvironment and low immunogenicity of the resected tumor site increase the risk of cancer recurrence and metastasis.^[^ ^23^ ^]^ Reprogramming the immunosuppressive microenvironment and enhancing the immunotherapy response are potential strategies to prevent tumor recurrence and prolong patient survival.^[^ ^24^ ^]^ The observed mitigation of tumor recurrence in OSCC mouse models after application of photoirradiated APHP‐CCCA hydrogel prompted us to investigate its immunomodulatory potential in low‐immunogenic OSCCs. On days 3 and 15 following implantation, we assessed immune cell responses in tumor tissues, lymph nodes, and typical immune cytokines in serum (Figure 3A).

**Figure 4:**
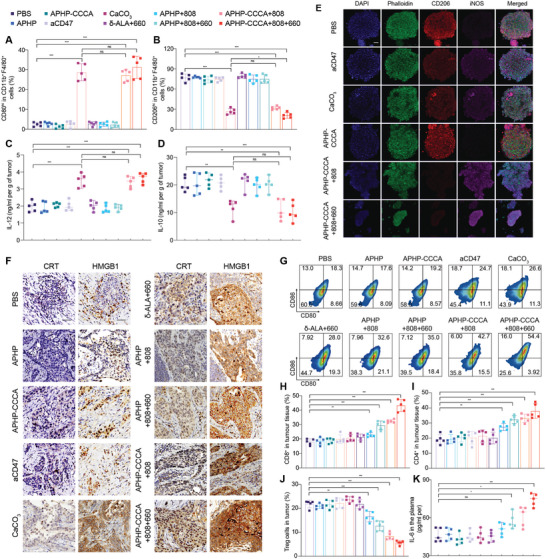
In vivo immunomodulatory effects of APHP‐CCCA hydrogel implantation postsurgery in an OSCC model. A,B) Relative quantification of flow cytometry analysis identifying M1‐like TAMs (CD80^hi^CD11b^+^F4/80^+^) and M2‐like TAMs (CD206^hi^CD11b^+^F4/80^+^) gated on F4/80^+^CD11b^+^ cells in residual OSCC tumor tissues obtained from mice in each group on day 3 following implantation. C,D) Quantitative ELISA analysis of IL‐12 (C) and IL‐10 (D) levels in residual OSCC tumor tissues obtained from mice in each group on day 3 after hydrogel implantation. E) Representative immunofluorescence images showing the polarization of macrophages from the M2 to the M1 phenotype following different treatment. Red: M2 marker (CD206); purple: M1 marker (iNOS); blue: DAPI; green: phalloidin. Scale bar: 20 µm. F) Immunohistochemical staining detectingCRT expression and subcellular localization of the damage‐associated molecular pattern protein HMGB1 in residual tumor tissues obtained from mice in each group on day 3 after implantation. Scale bar, 50 µm. G) Representative flow cytometry analysis enumerating CD80^+^CD86^+^ mature DCs in the tumor‐draining lymph nodes of mice in each group on day 15 after implantation. H–J) Relative quantifications of the proportions of infiltrating CD8^+^ cytotoxic T cells (H) CD4^+^ T cells (I) and immunosuppressive Tregs (CD3^+^CD4^+^Foxp3^+^) (J) in residual tumor tissues obtained from mice in each group on day 15 after implantation. K) Quantitative ELISA analysis of IL‐6 levels in residual tumor tissues obtained from mice in each group on day 15 after implantation. Data are presented as the mean ± SD; *n* ≥ 3 independent experiments. *P* values were determined by two‐way ANOVA, Tukey's multiple‐comparison test (ns, not significant; ^*^ *p* < 0.05; ^**^ *p* < 0.01; ^***^ *p* < 0.001).

As CaCO_3_ nanoparticles released by APHP‐CCCA hydrogel could scavenge H+ and induce M2 to M1 polarization of tumor‐associated macrophages (TAMs),^[^ ^20^ ^]^ we examined the proportion of TAMs in tumor tissues. Only the “CaCO_3_”, “APHP‐CCCA+808”, and “APHP‐CCCA+808+660” groups demonstrated reduction of M2‐like TAMs (CD206^hi^CD11b^+^F4/80^+^) and an increase in M1‐like TAMs (CD80^hi^CD11b^+^F4/80^+^) on day 3 after implantation (**Figure** **4**A–B). This polarization was further confirmed by the increased level of interleukin‐12 (IL‐12, the predominant cytokine secreted by M1‐like TAMs) (Figure 4C) and the reduced level of interleukin‐10 (IL‐10, the predominant cytokine secreted by M2‐like TAMs) (Figure 4D) in the tumor microenvironment (TME) of the three groups. In the “CaCO_3_” group, M1‐like TAMs increased, and M2‐like TAMs decreased on day 3; however, these effects were not sustained on day 15 (Figure S6A,B, Supporting Information). As free drug diffuses away, it cannot maintain sufficient concentration at the tumor site, resulting in diminished therapeutic effects and early recurrence. Immunofluorescence analysis demonstrated that aCD47 and CaCO_3_ nanoparticles released by APHP‐CCCA effectively induced the polarization of macrophages from the M2 to the M1 phenotype in vitro (Figure 4E) and enhanced the phagocytic activity of macrophages against cancer cells in vitro (Figure S6C, Supporting Information). Consequently, the APHP‐CCCA‐induced shift from M2 to M1 macrophages, coupled with CD47 blockade, facilitated the phagocytosis of cancer cells, thereby activating the innate immune response.

We continued to evaluate whether PTT, PDT and CIT treatments could induce ICD and stimulate antitumor immunity in vivo. ICD is a unique form of cell death that can release immunogenic damage‐associated molecular patterns (DAMPs) and tumor‐associated antigens, triggering long‐term protective antitumor immune responses.^[^ ^25^ ^]^ The expression levels of calreticulin (CRT) and subcellular localization of high mobility group box 1 (HMGB1) in tumor tissues after different treatments were analyzed by immunohistochemical staining (Figure 4F; Figure S6D–H, Supporting Information). On day 3 postimplantation, an obvious increase in CRT expression and cytoplasmic translocation of nuclear HMGB1 was observed in the “APHP‐808”, “δ‐ALA+660”, and “CaCO_3_” groups (Figure 4F; Figure S6E,F, Supporting Information), indicating that PTT, PDT, and CIT can induce ICD in vivo. On day 15 postimplantation, CRT expression and HMGB1 cytoplasmic translocation were much higher in the “APHP‐CCCA+808+660” group compared to other groups (Figure S6D,G,H, Supporting Information), demonstrating that APHP‐CCCA‐mediated in vivo phototherapy could efficiently elicit ICD. The apparent absence or inadequate staining in the central region of several tongue tumors is attributed to liquefactive necrosis occurring within the internal tissue of the tumor.

Matured DCs can present tumor antigens to T cells and trigger subsequent immune responses.^[^ ^26^ ^]^ Flow cytometry results demonstrated that the proportion of mature DCs (CD11c^+^CD80^+^CD86^+^) in the tumor draining lymph nodes (the main organ for DC maturation) was highest in the “APHP‐CCCA+808+660” group (Figure 4G; Figure S7A, Supporting Information) on day 15. Flow cytometry analysis also showed the percentage of tumor‐infiltrating CD8^+^ T cells, an indicator of antitumor immunity activation, was 2.40‐fold higher in the “APHP‐CCCA+808+660” group compared to the PBS group (Figure 4H). This value increased to 1.89‐fold in the “APHP+808” group, 1.47‐fold in the “APHP+808+660” group, and 1.34‐fold in the “APHP‐CCCA+808” group, demonstrating the exceptional immunostimulatory effect of the synergistic combination of photoimmunotherapy and immunomodulatory agents (aCD47 and CaCO_3_) (Figure 4H). Consistently, CD4^+^ T cells in tumors were significantly improved in mice receiving “APHP‐CCCA+808+660” treatment compared to all other groups (Figure 4I) on day 15.

We next examined the levels of tumor regulatory T cells (Tregs, CD3^+^CD4^+^Foxp3^+^), which can suppress the antitumor immune responses of cytotoxic T lymphocytes and induce an immunosuppressive microenvironment.^[^ ^27^ ^]^ The results demonstrated that the Tregs frequency in the “APHP‐CCCA+808+660” group was 3.5‐fold lower than in the PBS group on day 15, indicating effective reduction of tumor‐associated immunosuppression (Figure 4J). Mice in the “APHP‐CCCA+808+660” group showed significantly increased levels of typical immune‐related cytokines in plasma, including interleukin 6 (IL‐6) (Figure 4K), interferon γ (IFN‐γ) (Figure S7B, Supporting Information), tumor necrosis factor α (TNF‐α) (Figure S7C, Supporting Information), and interleukin 2 (IL‐2) (Figure S7D, Supporting Information), all contributing to activation of systemic T‐cell response. These results revealed the in situ vaccine hydrogel can efficiently reprogram the immunosuppressive tumor microenvironment and induce ICD in vivo, thereby enhancing immune stimulation.

### 2.7 Abscopal Effect of APHP‐CCCA in Inhibiting Distant OSCC Tumors

**Figure 5:**
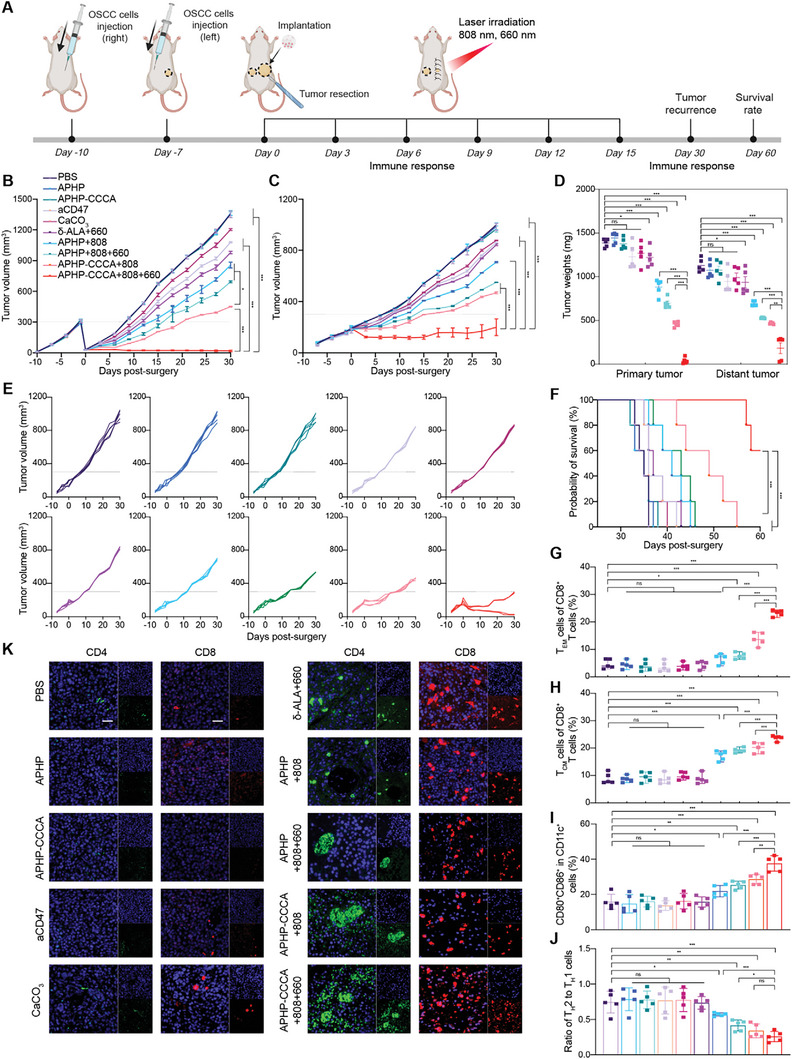
Abscopal antitumor effects induced by APHP‐CCCA hydrogel implantation postsurgery in OSCC tumors. A) Schematic diagram of the experimental design. Balb/c mice were inoculated subcutaneously with SCC7 OSCC cells into the right flank. Three days later, mice were inoculated with SCC7 cells into the left flank to establish distant secondary tumors. Mice then underwent surgical resection of the primary right flank tumor followed by implantation of hydrogels or control treatments at the tumor resection site, followed by 6 rounds of NIR laser irradiation at the primary tumor site. NIR irradiation parameters: 808 nm laser at 1.25 W cm^−2^ for 5 min and/or 660 nm laser at 40 mW cm^−2^ for 5 min. B,C) Average growth curves of recurrent primary tumors (B) and untreated distant secondary tumors (C) in the different experimental groups over 30 d following primary tumor resection and hydrogel implantation. Data shown as mean ± SEM (*n* = 5 mice per group). D) Weights of excised primary recurrent tumors (left) and distant secondary tumors (right) from each experimental group at day 30 postsurgery. (*n* = 5 mice per group). E) Individual distant secondary tumor growth curves of mice in each experimental group over 30 d following surgery and hydrogel implantation at the primary site. F) Kaplan–Meier survival curves showing the percentage of mice surviving over 60 d following tumor resection and hydrogel implantation (*n* = 5 mice per group). G,H) Representative quantification of flow cytometry analyses of systemic antitumor immune responses in PBMCs isolated from mice in each experimental group. T_EM_ (CD62L^−^CD44^+^CD8^+^) (G) and T_CM_ (CD62L^+^CD44^+^CD8^+^) (H) were quantified at the day 6 after hydrogel implantation. I) Representative quantification of flow cytometry analyses of systemic antitumor immune responses in PBMCs isolated from mice in each experimental group. Mature DCs (CD11c^+^CD80^+^CD86^+^) were quantified at the day 30 after hydrogel implantation. J) Representative flow cytometry quantification of the ratio of T_H_2 (CD45^+^CD4^+^IL‐4^+^) to T_H_1 (CD45^+^CD4^+^IFN‐γ^+^) cells in PBMCs isolated from mice in each experimental group on day 30 after hydrogel implantation. K) Immunohistochemical staining detecting CD4^+^ (green) and CD8^+^ (red) T cell infiltration within distant secondary OSCC tumors obtained on day 30 postimplantation. Scale bar, 50 µm. Data are presented as the mean ± SD; *n* ≥ 3 independent experiments. *P* values were determined by two‐way ANOVA, Tukey's multiple‐comparison test (ns, not significant; ^*^ *p* < 0.05; ^**^ *p* < 0.01; ^***^ *p* < 0.001).

Occult metastases in OSCC are difficult to detect clinically, which is a major obstacle to cancer treatment and the leading cause of death.^[^ ^28^ ^]^ ≈30% of OSCC patients with negative lymph nodes have occult metastases that are undetectable by physical or radiological examination.^[^ ^29^ ^]^ We investigated whether in situ vaccination with APHP‐CCCA and multiple irradiation cycles could induce systemic antitumor immunity to treat distant tumors with an abscopal effect in Balb/c mice bearing contralateral SCC7 tumors. SCC7 cells (5 × 10^5^) were subcutaneously inoculated on the right flank of each Balb/c mouse as the primary tumor. Three days later, SCC7 cells (2 × 10^5^) were inoculated on the left flank of the same mouse as the distant tumor (**Figure** **5**A). Then, ≈90% of primary tumors (≈300 mm^3^) were surgically removed, mimicking incomplete resection of tumors in the clinic (Figure 5A). Hydrogels were implanted at the surgical resection site before suturing, followed by 6 rounds of laser irradiation, leaving distant tumors untreated.

The “APHP‐CCCA+808+660” group postponed the tumor growths of primary (Figure 5B) and distant tumors (Figure 5C) compared to PBS groups with inhibition rates of 86% and 83%, respectively. Similarly, “APHP‐CCCA+808+660” group outperformed other groups in the reduction of tumor weights in both primary and distant tumors (Figure 5D). “APHP‐CCCA+808+660” further potentiated the therapeutic efficacy of distant tumors, with 126%, 110%, and 30% increase of survival rate relative to “APHP+808”, “APHP+808+660”, and “APHP‐CCCA+808” groups (Figure 5F). Furthermore, 2 out of 5 distant tumors in the “APHP‐CCCA+808+660” group had complete regression and showed no signs of recurrence within 60 days of initial implantation (Figure 5E). Based on these results, it appears that in situ vaccination of APHP‐CCCA after surgery causes systemic tumor protection with an abscopal effect.

To dissect the systemic antitumor immunity underlying the above distant tumor inhibition, we analyzed peripheral blood mononuclear cell (PBMC) immune milieu on day 6 and 30 after implantation. The “APHP‐CCCA+808+660” group showed increased fraction of CD8^+^ effector memory T cells (T_EM_, CD62L^−^CD44^+^CD8^+^) and central memory T cells (T_CM_, CD62L^+^CD44^+^CD8^+^) on day 6 (Figure 5G,H), which was further enhanced with the laser irradiations cycle increased, with 5.1‐ and 4.7‐fold increases of relative to PBS group on day 30 (Figure S8A,B, Supporting Information). “APHP‐CCCA+808+660” further expanded the proportion of mature DCs (CD11c^+^CD80^+^CD86^+^) (Figure 5I) and decreased the ratio of T helper 2 (T_H_2, CD45^+^CD4^+^IL‐4^+^) to T helper 1 (T_H_1, CD45^+^CD4^+^IFN‐γ^+^) cells in PBMCs on day 30 (Figure 5J), indicating multitier systemic antitumor immunomodulation.^[^ ^30^ ^]^ On day 30 postimplantation, the increased infiltration of CD8^+^ and CD4^+^ T cells within the distant tumors of “APHP‐CCCA+808+660” group was further confirmed by immunofluorescence staining (Figure 5K; Figure S8C,D, Supporting Information). Serum secretion of cytokines encompassing IFN‐γ (Figure S8E, Supporting Information), IL‐6 (Figure S8F, Supporting Information), and IL‐12 (Figure S8G, Supporting Information) additionally substantiated the efficacious systemic antitumor immunomodulation provoked by “APHP‐CCCA+808+660”. These findings imply that in situ vaccination with APHP‐CCCA elicited a robust systemic antitumor immune response, culminating in an abscopal effect on distant tumors.

### 2.8 Abscopal Effect of APHP‐CCCA in Preventing Orthotopic OSCC and Lung Metastasis

**Figure 6:**
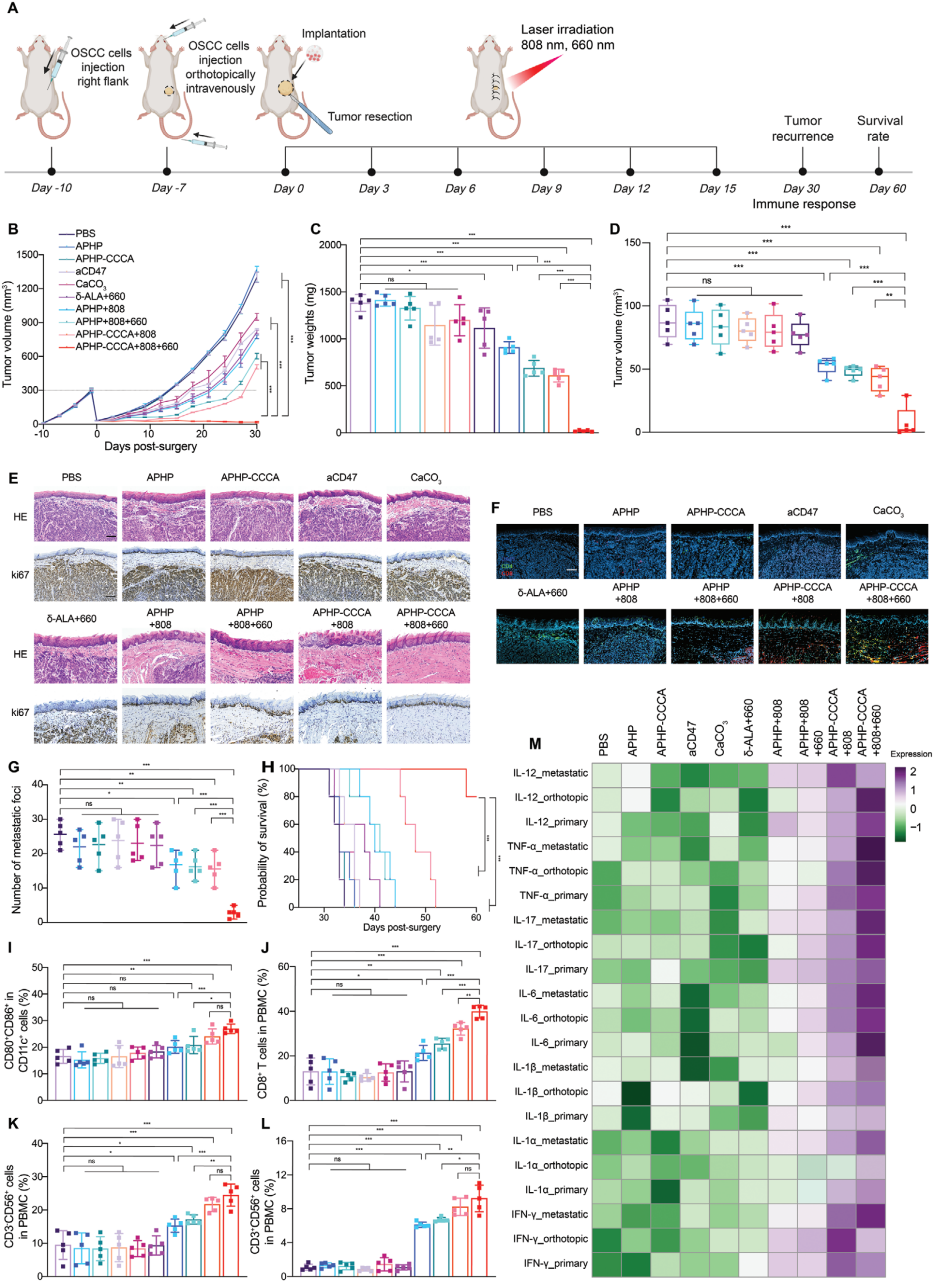
Abscopal effect of APHP‐CCCA in preventing orthotopic OSCC tongue tumors and lung metastasis. A) Schematic of the experimental design. Balb/c mice were inoculated subcutaneously with SCC7 cells into the right flank. Three days later, SCC7 cells were implanted orthotopically into the tongue, and a small number of SCC7 cells were infused via the tail vein to simulate hematogenous dissemination and lung metastasis. Mice underwent resection of the right flank tumor followed by hydrogel implantation at the site. NIR parameters: 808 nm at 1.25 W cm^−2^ for 5 min and/or 660 nm at 40 mW cm^−2^ for 5 min. B) Average growth curves of recurrent flank tumors in each group over 30 d postresection and hydrogel implantation. Data shown as mean ± SEM (*n* = 5 mice per group). C) Weights of recurrent flank tumors harvested on day 30 postsurgery and hydrogel implantation (*n* = 5 mice per group). D) Volumes of orthotopic tongue tumors in each group on day 30 postsurgery. (*n* = 5 mice per group). E) H&E and Ki67 immunohistochemical staining of orthotopic tongue tumors at day 30 postsurgery. Scale bars, 10 mm. F) Immunofluorescence staining showing CD4^+^ (green) and CD8^+^ (red) T cell infiltration in orthotopic tongue tumors at day 30 postsurgery. Scale bar, 10 mm. Blue: DAPI. G) Quantification of lung metastatic foci numbers per lung at day 60 postimplantation. H) Kaplan–Meier survival curves over 60 d post‐tumor resection and hydrogel implantation (*n* = 5 mice per group). I–L) Flow cytometry quantification of mature Dcs (CD80^+^CD86^+^ of CD11c^+^) (I), CD8^+^ T cells (J), NK cells (CD3^−^CD56^+^) (K), and NKT cells (CD3^+^CD56^+^) (L) in PBMCs at day 30 postimplantation. M) Heatmap of cytokine levels (IL‐12, TNF‐α, IL‐17, IL‐6, IL‐1β, IL‐1α, IFN‐γ) measured by ELISA in treated flank tumors, untreated orthotopic tongue tumors, and lung metastases at day 30. Data are presented as the mean ± SD; *n* ≥ 3 independent experiments. *P* values were determined by two‐way ANOVA, Tukey's multiple‐comparison test (ns, not significant; ^*^ *p* < 0.05; ^**^ *p* < 0.01; ^***^ *p* < 0.001).

Lung metastases are the most common distant metastases in OSCC, with an incidence of 70–85%.^[^ ^31^ ^]^ Up to 50% of patients with occult metastases from OSCC experience extracapsular spread.^[^ ^32^ ^]^ As a result of these tumor cells being shed from the primary tumor, they are termed “seeds” of lung metastatic disease. We then investigated whether in situ vaccination of APHP‐CCCA with multiple irradiation cycles for the treatment of orthotopic OSCC and the development of lung metastasis. The use of photoimmunotherapy offers a powerful and durable treatment for OSCC as well as the prevention of lung metastases by eliciting systemic antitumor immune responses. To mimic local and distant tumors in the case of lung metastasis, we established a multitumor mouse model with an orthotopic murine OSCC and a subcutaneous OSCC tumor on the flanks (**Figure** **6**A). SCC7 cells were subcutaneously injected (5 × 10^5^) onto the right flank. Three days later, SCC7 cells were orthotopically implanted (4 × 10^4^) into the tongues, and a small number of SCC7 cells (1 × 10^3^) were also infused into the tail vein to simulate the shedding of tumor cells into the bloodstream that may result in lung metastasis. Then, ≈90% of primary tumors (≈300mm^3^) were surgically removed and hydrogels were implanted at the surgical resection followed by 6 rounds of laser irradiation, leaving the orthotopic tumors untreated (Figure 6A).

Treatment with “APHP‐CCCA+808+660” significantly enhanced therapeutic efficacy against not only subcutaneous tumors (Figure 6B,C), but also orthotopic OSCC and lung metastases (Figure 6D–G), suggesting that in situ vaccination with APHP‐CCCA postsurgery may lead to abscopal effects in OSCC. Specifically, “APHP‐CCCA+808+660” induced regression of 3 out of 5 subcutaneous tumors (Figure 6B,C; Figure S9A, Supporting Information) and remarkably, complete regression of 4 out of 5 orthotopic OSCC tumors (Figure 6D,E). When analyzing tongue tissue collected on day 30 after implantation, no precancerous tumors were found in the tongues of the 4 mice in “APHP‐CCCA+808+660” group (Figure 6D,E), and Ki67 expression in the tongue tissue of this group was significantly lower than all other groups (Figure S9B, Supporting Information). Furthermore, analysis of whole lung tissues harvested on day 60 postimplantation revealed that the number of lung metastatic foci in the “APHP‐CCCA+808+660” group was remarkably lower than all other groups (Figure 6G), ≈15‐fold lower than the PBS group (Figure 6G). “APHP‐CCCA+808+660” further potentiated the therapeutic efficacy of orthotopic OSCC and lung metastases, with a median survival of 69 d compared to 33 d in the PBS group (Figure 6H). This suggests that the sustained and controlled release of δ‐ALA, aCD47, and CaCO_3_ through NIR‐responsive hydrogel degradation provided long‐term prevention of residual OSCC cell migration into the lungs.

We analyzed PBMC immune milieu on day 30 to investigate the systemic antitumor immunity underlying the abscopal effect of APHP‐CCCA for the treatment of orthotopic OSCC and lung metastases. Six‐cycle NIR of “APHP‐CCCA+808+660” increased the fractions of matured DCs by 4.3‐ and 4.6‐fold relative to PBS and “APHP‐CCCA” groups, respectively (Figure 6I). “APHP‐CCCA+808+660” augmented the natural killer (NK) cells (CD3^−^CD56^+^) (Figure 6K), natural killer T (NKT) cells (CD3^+^CD56^+^) (Figure 6L), and CD8^+^ T cell populations (Figure 6J), suggesting eliciting robust and durable innate and adaptive antitumor immunity to effectively suppress the distant orthotopic OSCC and lung metastases. These findings suggest that robust and durable antitumor immunity was elicited to suppress distant orthotopic OSCC and lung metastases effectively through both systemic innate (NK and NKT cells) and adaptive (CD8^+^ T cell) mechanisms.^[^ ^33^ ^]^

We further examined the impact of in situ vaccination APHP‐CCCA of primary tumors on the TME immune milieu in both orthotopic OSCC and distant lung metastases. The cytokine levels in the treated primary tumors, untreated orthotopic tumors, and distant lung metastases were determined by ELISA on day 30 (Figure 6M). Relative to PBS, “APHP‐CCCA+808+660” significantly increased the cytokine levels in treated tumors, orthotopic tumors, and lung metastases (IL‐12, TNF‐α, IL‐17, IL‐6, IFN‐γ, *p* < 0.05) (Figure 6M). Moreover, relative to “APHP‐CCCA” and “APHP‐CCCA+808”, “APHP‐CCCA+808+660” significantly increased CD8^+^ and CD4^+^ T cells level in untreated orthotopic tumors on day 30 (Figure 6F; Figure S9C,D, Supporting Information). Collectively, in situ tumor vaccination with APHP‐CCCA remodeled tumor immune milieu and promoted TME antitumor immunity not only in the treated primary tumors but also in orthotopic OSCC and distant lung metastases.

### 2.9 Long‐Term Immune Memory Effects of In Situ Vaccination with APHP‐CCCA

**Figure 7:**
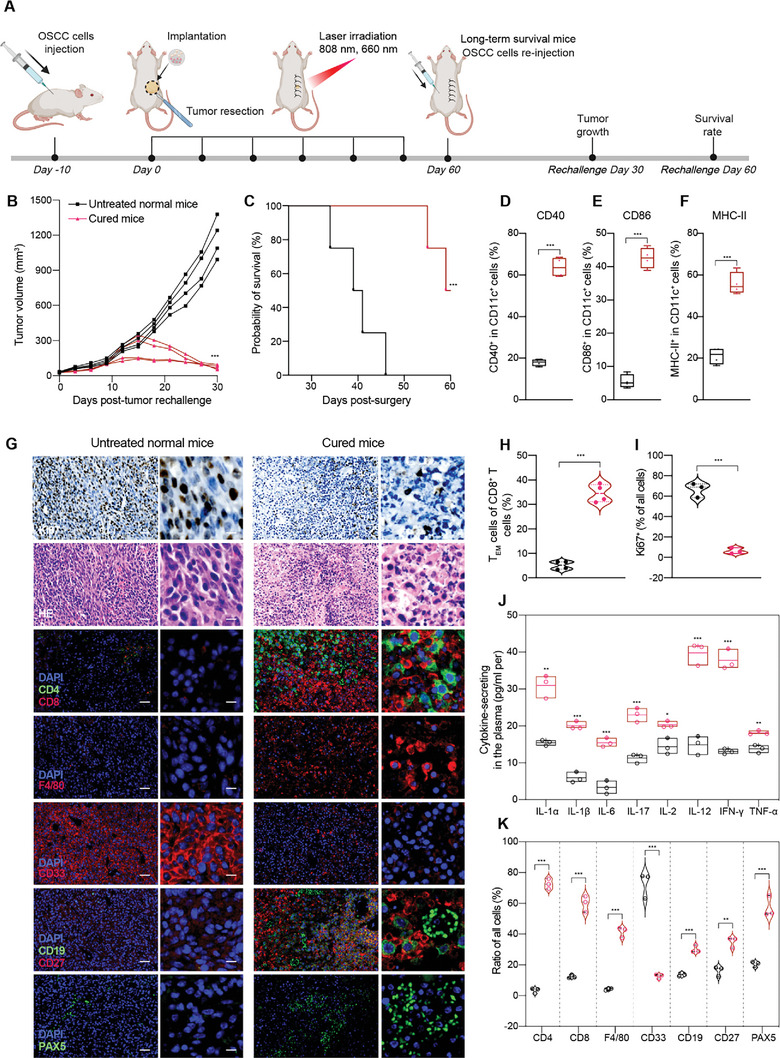
Durablec systemic antitumor immune memory effects elicited by in situ vaccination with APHP‐CCCA hydrogel. A) Schematic overview of the experimental design. Mice exhibiting long‐term survival following intraoperative implantation of APHP‐CCCA hydrogel and near‐infrared photoimmunotherapy (“APHP‐CCCA+808+660”) were rechallenged with subcutaneous injection of SCC7 cells into the left flank on day 60 posthydrogel treatment. As controls, untreated Balb/c mice received SCC7 challenge. B) Individual tumor growth curves of “APHP‐CCCA+808+660” cured mice compared to untreated naive control mice over 30 d following SCC7 tumor rechallenge. C) Kaplan–Meier survival analyses over 60 d after SCC7 tumor reinoculation (*n* = 4 mice per group). D–F) Flow cytometric quantification of DC maturation markers CD40 (D), CD86 (E), and MHC class II (F) expression on CD11c^+^ DCs isolated from the PBMCs of long‐term cured mice and untreated naive controls. G) Representative H&E staining and Ki67 immunohistochemistry of rechallenged tumors at 30 d postreinoculation (scale bars, 10 mm and 10 µm). Representative fluorescence images showing infiltration of CD4^+^ and CD8^+^ T cells, F4/80^+^ macrophages, CD33^+^ myeloid cells, CD19^+^ B cells, CD27^+^ memory T and B cells, and PAX5^+^ B cells within rechallenged tumors of cured mice and untreated controls (scale bars, 10 mm and 10 µm). H) Flow cytometric quantification of T_EM_ cells isolated from PBMCs of long‐term cured mice and untreated naive control mice. I) Relative quantification of immunohistochemical staining detecting Ki67 expression within rechallenged tumors of cured mice and untreated controls. Scale bars, 20 mm. J) Quantitative ELISA analysis of cytokine levels in serum obtained from cured mice and untreated controls on day 15 post‐tumor rechallenge. (K) Quantification of representative immunofluorescence images showing infiltration of multiple immune cell subsets within rechallenged tumors of cured mice compared to untreated controls. Data are presented as the mean ± SD; *n* ≥ 3 independent experiments. *p* values were determined by two‐way ANOVA, Tukey's multiple‐comparison test (ns, not significant; ^*^ *p* < 0.05; ^**^ *p* < 0.01; ^***^ *p* < 0.001).

In adaptive immune response, immunological memory plays an important role in fighting diseases when the same types of pathogens invade again.^[^ ^34^ ^]^ It is thus possible for cancer vaccines to provide a long‐term immune‐memory effect that could prevent cancer recurrence.^[^ ^14^, ^35^ ^]^ To examine whether in situ vaccination APHP‐CCCA could induce a memory response, mice with long‐term survival from “APHP‐CCCA+808+660” group were rechallenged with SCC7 cells (5 × 10^5^) on day 60 to determine whether new tumors can be developed on the left flank, and untreated normal Balb/c mice were challenged as control (**Figure** **7**A).

Tumor can be clearly observed in untreated normal Balb/c mice on day 7 after OSCC cell inoculation and continued to grow into large tumors in the next 3 weeks (Figure 7B). The growth of the rechallenged SCC7 tumors on the four cured mice in “APHP‐CCCA+808+660” group were obviously suppressed over the 30‐d period (Figure 7B). Although some initial tumor growth was observed in 2 mice of the “APHP‐CCCA+808+660” group on day 15, the rechallenged SCC7 tumor was not detectable on day 30, suggesting that there was effective inhibition of tumor growth in those surviving mice (Figure 7B). The overall survival of the mice in “APHP‐CCCA+808+660” group was significantly higher compared with the survival of the control group, with the median survival time of 63 d in contrast to 40 d of untreated normal Balb/c mice (Figure 7C). Immunohistochemical staining revealed decreased expression of the proliferation marker Ki67 in rechallenged tumors on day 15 following inoculation compared to tumors in normal mice (Figure 7G,I).

To study the immune status underlying the resistance to tumor rechallenge in the above mice, 15 d after rechallenging, we observed the enhanced expression of CD40 (Figure 7D), CD86 (Figure 7E), and MHC‐II (Figure 7F) on DCs from PBMCs of “APHP‐CCCA+808+660” group. The percentage of T_EM_ cells in the cured mice of “APHP‐CCCA+808+660” group was 2.4 folds higher than in the untreated normal Balb/c mice, which could provide durable protections against tumor cell rechallenge (Figure 7H). “APHP‐CCCA+808+660” also enhanced the fraction of circulating cytokine‐secreting, which contributed to the protective immunity against tumor rechallenge (Figure 7J). In addition, immunofluorescence staining of the rechallenged tumors on the 14th day following inoculation compared to the normal mice showed significantly increased infiltrations of both CD8^+^ T cells and CD4^+^ T cells (Figure 7G,K), which play critical roles in tumor immune surveillance and long‐term immune memory.^[^ ^36^ ^]^ After “APHP‐CCCA+808+660” treatment, both CD8^+^ and CD4^+^ T cells proliferate and differentiate into memory T cells specific for tumor antigens. The synergistic effects of CD8^+^ and CD4^+^ T cells help establish stable antitumor immune memory for sustained immunosurveillance.^[^ ^36^ ^]^

To thoroughly evaluate the immunomodulatory effects of “APHP‐CCCA+808+660” treatment on T cells, we conducted a battery of assessments on isolated murine CD4^+^ and CD8^+^ lymphocytes with magnetic beads form untreated normal mice and “APHP‐CCCA+808+660” treatment cured mice. Multiple functional assays decisively demonstrated enhanced activation and effector function following treatment: Cytokine analysis revealed increased secretion of inflammatory mediators IFN‐γ, TNF‐α, and IL‐2, indicating augmented CD4^+^ T cell activity (Figure S10A, Supporting Information). CCK‐8 proliferation assays exhibited substantially heightened expansionary capacity of both CD4^+^ and CD8^+^ T cells (Figure S10B,C, Supporting Information). Coculture cytotoxicity experiments highlighted elevated tumor cell apoptosis induction by CD8^+^ cytotoxic T lymphocytes through enhanced lactate dehydrogenase release (Figure S10D, Supporting Information). Additionally, RT‐qPCR quantification verified significant downregulation of multiple transcriptional checkpoints including PD‐1, TIM‐3, Lag‐3, substantiating alleviation of exhaustion to renew T cell immunocompetence (Figure S10E,F, Supporting Information). This panel of comprehensive evaluations confirms “APHP‐CCCA+808+660” treatment stimulates multifaceted reinvigoration of T lymphocyte responsiveness while simultaneously blocking onset of immunosuppressive exhaustion. Rekindling systemic antitumor immunity is achieved through coordinated stimulation of proliferation, cytotoxicity, and sustained functionality – effectively priming dynamic T cell participation to combat recurrence.

The infiltration of TAMs increased, as evidenced by the elevated expression of F4/80 on TAMs (Figure. 7G,K). The inhibitory myeloid‐derived suppressor cells decreased, with CD33 expression maintaining at a relatively low level (Figure 7G,K). The numbers of CD19^+^ B lymphocytes, CD27^+^ memory T and B cells increased (Figure 7G,K), which favors the generation of memory lymphocytes and antibody response.^[^ ^37^ ^]^ PAX5, a key transcription factor for B cells,^[^ ^38^ ^]^ also showed significantly upregulated expression (Figure 7G,K), indicating B cell activation. It has been demonstrated that photo‐irradiated APHP‐CCCA hydrogel was effective in inhibiting tumor recurrence and metastasis by producing a tumor‐specific long‐term memory immunity effect.

We also conducted RT‐qPCR analysis to investigate molecular distinctions among untreated tumors, recurrent tumors postsurgery, and tumors treated with our hydrogel vaccine. Preliminary findings reveal notable differences in gene expression patterns associated with tumor growth, immune evasion, and microenvironment interactions (Figure S11, Supporting Information). Compared to primary tumors, recurrent lesions exhibit accelerated proliferation signaling (c‐Myc, Cyclin D1), heightened immunosuppression markers (PD‐L1, IDO1), and a more immunosuppressed, vascularized stroma (VEGFR, TGF‐β). In contrast, hydrogel‐treated tumors demonstrate inhibited growth, immunosuppression, and stroma genes, along with enriched cytotoxicity markers (Granzyme B, IFN‐γ). These results suggest a distinct molecular signature associated with the hydrogel vaccine's impact on tumor dynamics, emphasizing its potential in modulating key pathways related to growth, immune response, and the tumor microenvironment.

### 2.10 Biosafety Assessment of In Situ Vaccination with APHP‐CCCA

The biocompatibility of implantable materials is an important requirement for their use in in vivo biological applications. Using hyaluronic acid and calcium alginate as the matrix, our hydrogel is highly bioavailable.^[^ ^39^ ^]^ Calcium alginate is an easily absorbed calcium supplement,^[^ ^40^ ^]^ whereas hyaluronic acid can be completely absorbed and metabolized by the body,^[^ ^41^ ^]^ which dispelled the concern on the biocompatibility of in situ vaccination with APHP‐CCCA postsurgery. To test the cytotoxicity of APHP‐CCCA on HOK cells, different concentrations of APHP‐CCCA leaching solution were used (Figure S12A, Supporting Information). Without NIR, the APHP‐CCCA exhibited no cytotoxicity to normal cells, which indicates that it has a good biosafety profile (Figure S12A, Supporting Information). In the normal tissues surrounding the implantation site, H&E images revealed that there was no chronic inflammatory reaction after 4 weeks of treatment (Figure S12B, Supporting Information). Moreover, no obvious damage or inflammation was seen on the H&E images of main organs in any of the groups (Figure S13, Supporting Information), and the body weight showed no difference between the groups (Figure S12C, Supporting Information).

The rapid dissemination of free molecular immunostimulants into the blood circulation, also known as cytokine storm syndrome, not only compromises their therapeutic efficacy but also raises safety concerns.^[^ ^42^ ^]^ Further, as CD47 is highly expressed on red blood cells and platelets, hematotoxicity has been a major concern among the various side effects observed in clinical trials of anti‐CD47 therapies.^[^ ^43^ ^]^ We then implanted free aCD47 in situ, injected aCD47 intravenously, and implanted APHP‐CCCA in situ using NIR at 660 and 808 nm (APHP‐CCCA+808+660), and measured serum levels of a panel of cytokines and chemokines (Figure S14A, Supporting Information), as well as performed whole blood assays (Figure S14B–E, Supporting Information) and serum biochemical assays (Figure S15A–H, Supporting Information) 6 h after implantation. In “APHP‐CCCA+808+660” group, the serum levels of IL‐1β, IL‐6, TNF‐α, CCL2, and MCP‐1 were significantly reduced 6 h after treatment, suggesting improved systemic tolerability, which could prevent immune toxicity caused by overly strong systemic inflammation (Figure S14A, Supporting Information). The complete blood panel test illustrated that “APHP‐CCCA+808+660” did not cause significant changes to the key hematological parameters and showed potential relief of hematotoxicity after implantation (Figure S14B–E, Supporting Information). The serum biochemistry assays demonstrated that “APHP‐CCCA+808+660” did not elicit appreciable variations in the fundamental serum biochemical indices following implantation (Figure S15A–H, Supporting Information). Taken together, these findings substantiate that APHP‐CCCA possesses favorable biocompatibility and negligible toxicity for in vivo applications.

## 3 Discussion

OSCC exhibits a high propensity for locoregional recurrence and distant metastasis, persisting despite extensive surgical resection and adjuvant therapy. The primary driver of recurrence lies in the proliferation of residual tumor cells surrounding the initial tumor site, which surgical removal often fails to completely eliminate. This study addresses the need for a targeted postoperative strategy to combat residual OSCC cells, aiming to induce immunogenic antitumor responses through the integration of immunotherapy and phototherapy within the resection cavity. The developed in situ vaccine, named APHP‐CCCA, features a dual crosslinked nanocomposite hydrogel encompassing a photothermal polydopamine‐hyaluronic acid matrix, photosensitizer‐loaded PLGA nanoparticles, immunomodulatory aCD47, and CaCO_3_ nanoparticles. Controlled release of these components facilitates spatiotemporally regulated cytotoxic phototherapy, calcium overload‐induced ICD, and remodeling of the immune microenvironment. In OSCC models, APHP‐CCCA implantation in the tumor resection cavity resulted in the eradication of residual cancer cells through PTT/PDT ablation and apoptosis induced by calcium interference. Local delivery of aCD47 promoted phagocytosis without causing systemic toxicity. Phototherapy and calcium overload induced ICD, releasing tumor antigens, DAMPs, and inflammatory mediators. This sustained in situ vaccination prompted systemic cytotoxic T cell immunity, establishing long‐term immunological memory and safeguarding against OSCC rechallenge. Treated mice exhibited no local recurrence, abscopal tumor suppression, and inhibition of lung metastases. This in situ vaccination strategy holds promise as a postoperative approach for managing residual OSCC by orchestrating the cytotoxic elimination of tumor cells and eliciting immunogenic antitumor responses, thus overcoming the limitations of systemic immunotherapies and ultimately preventing recurrence.

The partial efficacy of surgical tumor resection necessitates the use of adjuvant chemotherapy and radiotherapy to eliminate residual tumor cells.^[^ ^4a^ ^]^ However, the limited effectiveness of systemic therapies poses challenges,^[^ ^2^, ^8^ ^]^ prompting exploration into localized postoperative implantation of antitumor agents directly into the tumor resection cavity for more targeted prevention of tumor recurrence.^[^ ^44^ ^]^ Biomaterial carriers may play a crucial role in allowing encapsulation and localized delivery of antitumor treatments while maintaining drug stability and bioactivity, which is especially critical given that direct implantation of small molecule drugs or antibody therapies into the resection cavity could lead to rapid metabolism and clearance.^[^ ^45^ ^]^ In our work, hyaluronic acid and calcium alginate hydrogels were selected as carriers based on their capacity for sustained and controlled release kinetics, which can mitigate inflammation while promoting wound healing and enable prolonged antitumor efficacy.

To eradicate residual tumor cells following surgical resection, we employed NIR laser irradiation at 808 and 660 nm in conjunction with an implanted photothermal effector and photosensitizer. Prior studies have demonstrated a synergistic antitumor effect with the combination of PTT and PDT, whereby PTT increases tumor oxygenation, thus enhancing the efficacy of PDT, while PDT improves laser absorption by photothermal agents.^[^ ^46^ ^]^ Our findings, which exhibited enhanced apoptosis of OSCC cells following sequential irradiation with NIR lasers at 808 and 660 nm, provide support for this synergistic interaction between PTT and PDT. Both PTT and PDT are capable of eliciting ICD, effectively converting the residual tumor tissue into an in situ vaccine source of tumor antigens. Mice treated with the in situ hydrogel‐based PTT/PDT vaccination displayed sustained antitumor immunological memory and were protected against tumor rechallenge for an extended period. This enduring immunity arises from the generation of a specific antitumor immune response and the immunomodulatory effects associated with local vaccination within the prior tumor site. These effects include enhanced DC maturation, increased infiltration of cytotoxic T lymphocytes and NK cells, polarization of TAMs tumor‐associated macrophages toward an M1 phenotype, and the promotion of long‐lived immunological memory against tumor cells.^[^ ^47^ ^]^

CD47 has emerged as a promising therapeutic target in cancer due to its widespread overexpression across numerous malignancies.^[^ ^48^ ^]^ CD47 binds to SIRPα on macrophages and delivers a “don't eat me” signal, allowing cancer cells to evade phagocytic destruction. By blocking the CD47‐SIRPα axis, aCD47can promote the phagocytosis of tumor cells by macrophages.^[^ ^49^ ^]^ CD47 antibody therapies are now under evaluation in clinical trials across a range of cancer types.^[^ ^48^, ^50^ ^]^ However, on‐target toxicity poses a key challenge in the development of aCD47.^[^ ^50^ ^]^ Systemic anti‐CD47 blockade can induce the phagocytic destruction and agglutination of healthy red blood cells along with tumor cells, leading to unsafe hematologic side effects like anemia and thrombocytopenia.^[^ ^48^ ^]^ In this study, calcium alginate hydrogels loaded with aCD47 were implanted locally into the tumor resection cavity. Controlled hydrogel degradation with gradual antibody release could sustain macrophage phagocytosis of residual tumor cells, while avoiding systemic toxicity to normal blood cells. Local delivery of aCD47 via biomaterial carriers represents a promising approach to avoid on‐target/off‐tumor effects and improve the safety and efficacy of this novel cancer immunotherapy.

In situ hydrogel vaccination holds particular clinical promise for tumors located in superficial anatomic sites, such as head and neck, skin, or breast malignancies.^[^ ^51^ ^]^ The accessibility of these superficial tumors allows direct localized implantation of hydrogel vaccines into the tumor resection cavity.^[^ ^52^ ^]^ For superficial malignancies, achieving adequate drug exposure in the tumor while avoiding systemic toxicity presents a major challenge for standard postoperative adjuvant therapies like chemotherapy and radiotherapy.^[^ ^53^ ^]^ By enabling highly targeted treatment, in situ vaccination can concentrate cytotoxic and immunomodulatory effects within the tumor site while minimizing side effects.^[^ ^54^ ^]^ Additionally, the strong immunogenic stimuli generated within the vaccination site located in superficial tissues may promote superior activation of tumor antigen presenting cells and interactions with lymphocytes compared to deep‐seated tumors.^[^ ^55^ ^]^ Furthermore, the initiation of immune memory in superficial sites may enhance surveillance against metastasis to regional lymph nodes.^[^ ^55^ ^]^ Overall, the ability to directly access the tumor resection cavity makes in situ hydrogel vaccination exceptionally well‐suited to managing residual disease burden and preventing recurrence in superficial malignancies.

To enhance the translational potential of postoperative in situ implantation vaccines, some enhancements may be made to the hydrogel‐based vaccine formulation. The photoimmunotherapy preparations utilized in our study are characterized by their precise control over time and space, as well as their noninvasive and efficient nature.^[^ ^56^ ^]^ These preparations, known as PTT, PDT, and CIT, primarily consist of multicomponent materials. However, the preparation procedures for these materials are intricate, and they require stimulation from light sources of two distinct wavelengths for PTT and PDT. Consequently, these preparations often encounter challenges related to stability and operational complexity.^[^ ^16^, ^18^, ^51^ ^]^ Hence, the development of single‐component PTT/PDT preparations that may be activated by a solitary laser source has considerable importance.^[^ ^57^ ^]^ Moreover, the controllability of aCD47 release from the hydrogel may be enhanced by several means such as augmenting the crosslinking of the hydrogel network, increasing internal loading, or using external stimulation.^[^ ^15^, ^48^, ^50^ ^]^ Additional research is required to develop injectable hydrogels that can effectively showcase the advantageous outcomes of the proposed approach in models of metastatic and unresectable tumors.^[^ ^40^ ^b,^ ^58^ ^]^

Overall, the in situ hydrogel vaccination strategy developed in this work represents a promising postoperative therapeutic approach for managing residual tumor burden through combined direct cytotoxicity and immune stimulation. For OSCC patients, postoperative implantation of hydrogel vaccines loaded with photosensitizers and immunomodulators directly into the tumor resection cavity allowed dual photothermal and photodynamic ablation of residual tumor cells along with induction of antitumor immunological memory. More broadly, this novel in situ vaccination paradigm could transform surgical care across a wide array of solid malignancies by overcoming the inadequacies of systemic therapies for residual disease. With optimization, in situ vaccination promises improved outcomes by preventing recurrence and enabling long‐term survival.

## 4 Experimental Section

### Study Design

The objective of this study was to develop an implantable in situ vaccine hydrogel integrating phototherapy and immunotherapy to induce localized cytotoxicity against residual OSCC cells while reprogramming the tumor microenvironment to stimulate systemic antitumor immunity, preventing postoperative recurrence. We systematically evaluated the antitumor efficacy of the APHP‐CCCA hydrogel against postoperative OSCC recurrence. In all in vivo experiments, 6‐ to 8‐week‐old murine models were utilized, with 4–10 animals per group to ensure statistical power for distinguishing tumor progression and survival differences between groups. Mice were monitored for survival, tumor growth kinetics, and protective immunity against OSCC rechallenge. Sample sizes were determined based on previous experience. Animals were randomly assigned to groups matched by tumor volume and body weight. Investigators were not blinded during experiments and assessments. No data outliers were excluded. Mice were humanely euthanized upon impaired health or exceeding tumor volumes of 1500 mm^3^. Animals were sacrificed to analyze in situ tumor burden and immunomodulation. All experiments were performed in triplicate.

### Materials

Dopamine hydrochloride (C_8_H_11_NO_2_·HCl), fluorescein isothiocyanate (C_21_H_11_NO_5_S), *N*‐hydroxysuccinimide (C_4_H_5_NO_3_), 1‐(3‐dimethylaminopropyl)‐3‐ethylcarbodiimide hydrochloride (C_8_H_17_N_3_·HCl), dichloromethane (CH_2_Cl_2_), sodium hyaluronate, poly(lactic‐*co*‐glycolic acid) (PLGA), polyvinyl alcohol (PVA), and tris (hydroxymethyl) aminomethane hydrochloride (Tris‐HCl, C_4_H_11_NO_3_·HCl) were purchased from Macklin Biochemical Technology Co., Ltd (Shanghai, China). δ‐Aminolevulinic acid (δ‐ALA, C_5_H_9_NO_3_), calcium chloride (CaCl_2_), sodium chloride (NaCl), sodium carbonate (Na_2_CO_3_), 4‐(2‐hydroxyethyl)‐1‐piperazineethanesulfonic acid (HEPES, C_8_H_18_N_2_O_4_S), sodium hydroxide (NaOH), sodium alginate (C_5_H_7_O_4_COONa), and sodium periodate (NaIO_6_) were obtained from Aladdin Reagent Co., Ltd. (Shanghai, China). Anti‐CD47 antibodies (Biolegend, #127 518, clone miap301) were purchased from Biolegend. All chemical reagents were used as received without further purification.

### Synthesis of Dopamine Modified Hyaluronic Acid

Sodium hyaluronate (1.0 g) was dissolved in 50 mL of ultrapure water and stirred under nitrogen atmosphere for 2 h. Dopamine hydrochloride (235 mg) was then added, and the solution was stirred in the dark for 60 min, followed by addition of 1‐(3‐dimethylaminopropyl)‐3‐ethylcarbodiimide (EDC, 384 mg) and *N*‐hydroxysuccinimide (NHS, 214 mg). The reaction mixture was stirred at room temperature under low light for 12 h. The resultant products were transferred into a 3500 Da molecular weight cut‐off dialysis bag and dialyzed against frequently changed ultrapure water for 3 d. The dialyzed solution was subsequently lyophilized and stored protected from light prior to use.

### Synthesis of δ‐ALA@PLGA Microspheres

PLGA (100 mg) was dissolved in 1 mL of dichloromethane. To this solution, 100 µL of 0.05 mg µL^−1^ δ‐ALA was added. The mixture was emulsified by ultrasonication using a 500 W ultrasonic cell disruptor in cycles of 9 s on and 4 s off, repeated 3 times. The emulsion was then introduced into 10 mL of 1% PVA solution and further ultrasonicated at 900 W with the same on/off cycle. The ultrasonic emulsion was stirred at room temperature for 3 hours under a fume hood to allow solvent evaporation. The resulting δ‐ALA encapsulated PLGA microspheres were purified by repeated water washing, freeze‐dried, and stored at 4 °C until use.

### Synthesis of aCD47@CaCO_3_ Nanoparticles

Anti‐CD47 antibodies (aCD47, 100 µg) were conjugated to sodium alginate by first preparing a solution containing aCD47 (100 µg) in 1 mL of 50 × 10^−3^ m HEPES buffer (pH 7.1) with 140 × 10^−3^ m NaCl and 10 × 10^−3^ m Na_2_CO_3_. This solution was mixed with 1 mL of 1 × 10^−3^ m Tris‐HCl buffer (pH 7.6) containing 100 × 10^−3^ m CaCl_2_ and stirred at 4 °C for 12 h. The reaction mixture was then centrifuged at 14 800 rpm for 5 min to remove unconjugated antibodies, polymers and ions. The supernatant containing the aCD47‐conjugated alginate copolymers was collected and lyophilized for storage at −20 °C until further use.

### Preparation of Composite Gel APHP‐CCCA

Hydrogel composites were prepared using the following components: hyaluronic acid‐dopamine conjugate (HA‐DOPA, 20 mg), dopamine hydrochloride (DOPA, 100 mg), δ‐aminolevulinic acid encapsulated poly(lactic‐co‐glycolic acid) microspheres (δ‐ALA@PLGA, 40 mg), sodium alginate (30 mg), and anti‐CD47 antibody conjugated calcium carbonate nanoparticles (aCD47@CaCO_3_, 2 mg). The HA‐DOPA, DOPA, and δ‐ALA@PLGA were first dissolved in 1 mL of ultrapure water. The pH was then adjusted to 8–9 using 1 m NaOH solution, followed by addition of 10 mg of sodium periodate and stirring for 10 min to form a prepolymer solution. The solution was allowed to crosslink for 12 h to form a polydopamine hydrogel mold. The mold was soaked in ultrapure water for 24 h to remove residual sodium periodate. Separately, a 3% w/v sodium alginate solution was prepared by dissolving 30 mg of sodium alginate in 1 mL of ultrapure water. The aCD47@CaCO_3_ nanoparticles (2 mg) were dispersed in this solution. The composite hydrogel was prepared by slowly adding 200 µL of 10% w/v CaCl_2_ solution to the sodium alginate/nanoparticle dispersion in the polydopamine hydrogel mold and allowing crosslinking for 30 min.

### Cell Line

The human head and neck squamous cell carcinoma cell lines HN6 and Cal33 were obtained from the American Type Culture Collection (ATCC, Rockville, MD, USA). The human oral keratinocyte (HOK) cell line was acquired from the cell bank of the Chinese Academy of Sciences (Shanghai, China). The cells were cultured in Dulbecco's Modified Eagle's Medium supplemented with 10% fetal bovine serum, 100 units mL^−1^ penicillin and 100 µg mL^−1^ streptomycin at 37 °C in a humidified 5% CO_2_ atmosphere.

### Animals

Female Balb/c mice (6–8 weeks old, 18–20 g) were purchased from Jihui laboratory animal breeding Co., Ltd (Shanghai, China). All animals were housed in pathogen‐free facilities and maintained at 20±3 °C with 40–70% relative humidity and a 12 h light/dark cycle. The animal experimental protocols were approved by the Institutional Animal Care and Use Committee of Shanghai Jiao Tong University School of Medicine and performed in accordance with the Guide for the Care and Use of Laboratory Animals.

### Surface Plasmon Resonance (SPR)

To investigate the interaction between aCD47 and CD47, SPR analysis was performed using a Biacore biosensor equipped with a research‐grade CM5 sensor chip produced by GE Healthcare (Chicago, USA). Recombinant human CD47 protein was obtained from MedChemExpress (MCE, USA). Activation of the dextran surface was observed after introduction of a 1:1 mixture of NHS/EDC. The CD47 protein was immobilized on the CM5 chip via amine coupling at a concentration of 100 µg mL^−1^ in 10 × 10^−3^ m sodium acetate, pH 5.0. Solutions of aCD47 or differently treated hydrogel leaching solutions with protein concentrations ranging from 0.391 to 50 × 10^−9^ m in 8 gradients were prepared after determining protein concentration by BCA assay. Binding experiments were conducted under controlled conditions of temperature (25 °C) and buffer concentration (10 × 10^−3^ m HEPES buffered saline, pH 7.4). The flow rate was set to 50 µL min^−1^, and the association and dissociation processes were monitored for 60 and 300 s, respectively. Using the Biacore Evaluation software provided by GE Healthcare, the binding responses were linked to a 1:1 Langmuir binding model by nonlinear regression to determine the dissociation constant (*K*_D_).

### In Vivo Antitumor Efficacy

To evaluate the in situ postoperative antitumor efficacy of implanted hydrogels, subcutaneous xenografts were established to assess inhibition of tumor recurrence, bilateral xenografts and orthotopic as well as lung metastasis models were used to examine the abscopal effect.

### In Vivo Antitumor Efficacy: Subcutaneous Xenograft Experiment

To simulate incomplete tumor resection clinically, SCC7 oral cancer cells (5 × 10^5^) were inoculated in the right flank of mice. When the tumor volume reached ≈300 mm^3^, ≈90% of the tumor was surgically resected. SCC7 tumor‐bearing mice were randomly divided into 10 groups (*n* = 10 per group). Before suturing, the resection site of each mouse was treated with PBS, aCD47, CaCO_3_, δ‐ALA with 660 nm NIR (δ‐ALA+660), APHP hydrogel with no NIR, 808 nm NIR, or 808 nm and 660 nm NIR (APHP, APHP+808, APHP+808+660), APHP‐CCCA hydrogel with no NIR, 808 nm NIR, or 808 and 660 nm NIR (APHP‐CCCA, APHP‐CCCA+808, APHP‐CCCA+808+660). Mice treated with δ‐ALA+660, APHP+808, APHP+808+660, APHP‐CCCA+808, APHP‐CCCA+808+660 were irradiated with 660 nm (40 mW cm^−2^, 5 min) and/or 808 nm (1.25 W cm^−2^, 5 min) near infrared laser on postoperative days 0, 3, 6, 9, 12, and 15. Tumor volumes were measured every 3 days and calculated as: tumor volume = width^2^ × length × 0.5. On postoperative day 30, five mice per group were sacrificed for analysis of tumor residues. Survival time and body weight of the remaining five mice were monitored until 60 days after tumor resection. Major organs including heart, liver, lungs, spleen, and kidneys were collected for H&E staining. Blood was collected for whole blood and biochemical analysis.

### In Vivo Antitumor Efficacy: Abscopal Effect

Similar to the subcutaneous model, SCC7 cells (5 × 10^5^) were inoculated in the right flank of mice. After 3 d, SCC7 cells (2 × 10^5^) were inoculated in the left flank of the same mice. When the tumor reached ≈300 mm^3^, approximately 90% of the primary tumor in the right flank was surgically resected to simulate incomplete clinical resection. Mice were randomly divided into 10 groups and treated with corresponding hydrogels. This was followed by 6 cycles of laser irradiation, while the distal tumor in the left flank was not treated. To evaluate if repeated irradiation of APHP‐CCCA in situ could be used to treat primary OSCC and prevent lung metastasis, a multitumor mouse model was established. SCC7 cells (5 × 10^5^) were inoculated in the right flank, then after 3 d SCC7 cells (4 × 10^4^) were implanted orthotopically in the tongue, and a small number of SCC7 cells (1 × 10^3^) were injected into the tail vein to simulate cells entering circulation and potentially seeding lung metastases. ≈90% of the primary flank tumor (≈300 mm^3^) was then surgically resected concurrent with hydrogel implantation, followed by 6 cycles of laser irradiation. The orthotopic tumor was not treated. Tumor volumes were measured every 3 d. On post‐operative day 30, tumor residues were analyzed. Mouse survival time and body weight were monitored until 60 d after resection. Major organs were collected for H&E staining. Blood was collected for whole blood and biochemical analysis.

### ReChallenge Tumor Model and Assessment of Long‐Term Immune Memory

On post‐operative day 60, 4 completely cured mice from the “APHP‐CCCA+808+660” group and 4 healthy untreated Balb/c mice were inoculated with SCC7 cells (5 × 10^5^) in the left flank. Tumor volumes were measured every 3 d. On day 30 after reinoculation, tumor residues were analyzed. Mouse survival time and body weight were monitored until 60 d after reinoculation. Major organs were collected for H&E staining. Blood was collected for complete blood count and serum biochemistry.

### Rhod‐4 AM Staining for Calcium Influx

For the Rhod‐4 AM staining procedure, cells were plated overnight in growth medium at a density of 40 000 cells/well/100 µL for a 96‐well plate. Prior to staining, a Rhod‐4 stock solution was prepared by adding 20 µL of DMSO to the vial of Rhod‐4 and thoroughly mixing the components. Simultaneously, a 1× assay buffer was formulated by combining 9 mL of Hanks' Balanced Salt Solution (HHBS) with 1 mL of 10× Pluronic F127 Plus, followed by thorough mixing. The Rhod‐4 dye‐loading solution was then prepared by adding 20 µL of the Rhod‐4 stock solution into 10 mL of 1× assay buffer, resulting in a stable working solution for at least 2 h at room temperature. Subsequently, 100 µL/well of the Rhod‐4 dye‐loading solution was added to the cell plate. The dye‐loaded cells were incubated in a cell incubator for 30 min, followed by an additional 30 minutes of incubation at room temperature. To initiate the calcium flux assay, the Rhod‐4 dye‐loading solution was replaced with HHBS or other designated treatments to eliminate any residual probes. The assay was executed by monitoring the fluorescence intensity at Ex/Em = 540/590 nm using a fluorescence microscope.

### Isolation of Splenic Immune Cells and Induction Macrophage Polarization for 3D Coculture with SCC7 Cells

To isolate splenic immune cells from mice, the mice are first euthanized and their spleens are carefully harvested and placed in cold RPMI‐1640 medium. The spleens are then homogenized through a 70 µm cell strainer to obtain a single‐cell suspension, which is centrifuged at 300 × g for 5 min at 4 °C. Following centrifugation, red blood cells are lysed using RBC lysis buffer, and the remaining splenocytes are washed and resuspended in complete RPMI‐1640 medium. The adherent macrophages are then harvested and treated with IL‐4 (20 ng mL^−1^) for 48 h to induce M2 polarization. After polarization, the M2 macrophages are mixed with SCC7 cells at a 1:50 ratio and seeded at a density of 10 000 cells per well in low‐attachment 96‐well plates. The plates are centrifuged gently to facilitate the formation of 3D coculture spheroids, which are incubated at 37 °C in a 5% CO_2_ incubator for 24 h. Subsequently, the spheroids are treated with various agents to induce M2 to M1 macrophage polarization. The shift in macrophage phenotype is assessed using flow cytometry, staining for M2 markers (CD206) and M1 markers (iNOS), and further visualized through immunofluorescence.

### Fluorescence Microscopy Characterization of FITC‐Labeled Nanoparticles

Fluorescence microscopy imaging was conducted to characterize the FITC‐conjugated anti‐CD47 loaded calcium carbonate (FITC‐aCD47@CaCO_3_) nanoparticles. For microscopy sample preparation, thin slices of the hydrogel containing FITC‐aCD47@CaCO_3_ particles were sectioned to ≈0.5 mm thickness using a vibrating microtome. The sections were mounted onto glass slides and sealed under coverslips. Imaging was performed on an Olympus CKX53/U‐RFL‐T inverted epifluorescence microscope equipped with a mercury vapor lamp light source and FITC filter cubes (470–495 nm excitation; 510–550 nm emission). A 40× objective lens with a 0.75 numerical aperture was utilized for visualizing the fluorescent nanoparticles. Multiple random fields of view were selected across each sample to capture representative images. The microscope is coupled to an Olympus DP80 dual CCD camera for image acquisition. Native Olympus CellSens Dimension software was used to control the microscope, camera and illumination source. No additional processing or contrast enhancement algorithms were applied. For reference, a 10 µm scale bar is included, with the image showing bright fluorescent spots on an otherwise dark background, verifying the presence of the FITC‐labeled nanoparticles within the hydrogel.

### Flow Cytometry Analysis of Immune Cells

After corresponding treatments, the residual tumor and lymph nodes were surgically resected and PBMCs were collected. Cells were stained with fluorescence‐labeled antibodies. The FITC‐conjugated anti‐CD11b antibody (#557 396), PE‐conjugated CD11c antibody (#557 401), BB700‐conjugated F4/80 antibody (#746 070), PE‐Cy7‐conjugated CD86 antibody (#560 582), BV421‐conjugated CD80 antibody (#562 611), PE‐Cy7‐conjugated CD56 antibody (#557 747), BV510‐conjugated CD45 antibody (#563 891), FITC‐conjugated CD3 antibody (#561 827), BV605‐conjugated CD4 antibody (#563 151), and PE‐Cy5.5‐conjugated CD8 (#561 109) were purchased from BD Bioscience (USA). CD206 antibody (#141 708) and Foxp3 (#126 404), CD62L (#104 406), CD44 (#103 018), and IFN‐γ (#505 830), IL‐4 (#504 124) were obtained from Biolegend (USA). All antibodies were diluted according to the manufacturer's instructions. The stained cells were analyzed on a BD LSRFortessa flow cytometer (BD, USA) and the data were analyzed using FlowJo software. The numbers presented in the flow cytometry analysis images represent the percentage of positive cells.

### Statistical Analysis

Statistical analysis was performed using GraphPad Prism 8. Data are presented as mean ± s.d. Tukey post‐hoc tests were used for multiple comparisons (when more than two groups were compared), and Student's *t*‐test was used for two‐group comparisons. Survival was plotted using Kaplan–Meier curves and assessed using two‐sided log‐rank (Mantel–Cox) tests. The threshold for statistical significance was *p* < 0.05.

## Conflict of Interest

The authors declare no conflict of interest.

## Author Contributions

L.C., Q.Y., and H.Z. contributed equally to this work. S.S., X.C., and Y.Y. conceived the study and designed the experiments; Y.Y., L.C., Q.Y., and H.Z. wrote the manuscript. Q.Y., H.Z., L.W., and T.L. synthesized and characterized the hydrogels, and collected the data. Y.Y., L.C., J.Z., G.Y., C.X., P.X., J.C.Z., and H.Z. performed the animal experiments, conducted the in vitro experiments, and collected the data. All authors analyzed and interpreted the data, contributed to the writing of the manuscript, discussed the results and implications, and edited the manuscript at all stages.

## Supporting information



Supporting Information

## Data Availability

The data that support the findings of this study are available in the supplementary material of this article.
