# Supplementary material for: Protecting Against Postsurgery Oral Cancer Recurrence with an Implantable Hydrogel Vaccine for In Situ Photoimmunotherapy
Source: Adv Sci (Weinh). 2024 Oct 28;11(46):2309053. doi: 10.1002/advs.202309053 (PMC11633475; doi:10.1002/advs.202309053)
Supplement: Supplementary file 1 — Supporting Information [file ADVS-11-2309053-s001.docx]

**Materials and Methods**

***Cell couting kit 8 (CCK-8) assays***

The cell viability of HN6, Cal33, and HOK cells under various treatment conditions with and without laser irradiation was assessed using the CCK-8 assay kit (Beyotime, Shanghai, China) following the manufacturer's protocol. Briefly, the cells were seeded into 96-well plates at a density of 3,000 cells per well. After allowing cell adhesion, the cultures were subjected to the indicated treatments with or without laser irradiation for the specified time periods. At the end of the treatments, 10 μL of CCK-8 reagent was added to each well containing 100 μL of culture medium and incubated for 2 h at 37°C. The optical density at 450 nm was then measured using a Biotek Microplate Reader to determine cell viability.

***Reactive oxygen species (ROS) production***

HN6 and Cal33 head and neck squamous cell carcinoma cells were seeded in 12-well plates at a density of 20,000 cells per well and allowed to adhere overnight. The cells were then treated with various nanoparticles with or without simultaneous laser irradiation for 24 h. After treatments, the cells were incubated with 10 μM 2’,7’-dichlorofluorescin diacetate (DCFH-DA; Beyotime) diluted in DMEM for 20 minutes at 37°C. Subsequently, the cells were washed twice with PBS and resuspended in PBS. Fluorescence microscopy was utilized to analyze intracellular ROS production in the treated cells.

***Transmission electron microscopy assay (TEM)***

To assess mitochondrial damage and apoptosis after hydrogel treatment, the ultrastructural morphology of mitochondria in OSCC cells was observed by TEM assay. Briefly, the OSCC cultures were subjected to the indicated treatments with or without laser irradiation for the specified time periods, then fixed, dehydrated, embedded, sectioned, and stained using standard TEM sample preparation methods. Thin sections were examined under a Hitachi HT7800/HT7700 transmission electron microscope and images were captured. Mitochondrial ultrastructural changes such as swelling, vacuolization, and disruption of cristae were evaluated to determine mitochondrial injury and apoptosis. At least 100 cells from each group were assessed to quantify the percentage of cells exhibiting mitochondrial damage.

***Immunofluorescence staining***

Tumors were collected from the mice and snap frozen in optimal cutting temperature medium. Tumor sections were cut using a cryotome, mounted on slides and stained with different primary antibodies: CD4 (Servicebio, # GB15064-100), CD8 (Servicebio, # GB114196-100), F4/80 (Servicebio, # GB113373-100), CD33 (Thermo, # 14-0338-82), CD19 (Thermo, # 14-0199-82), CD27 (Abcam, # ab131254), and PAX5 (Abcam, #ab109443) overnight at 4 °C following the manufacturer's instructions. Following the addition of fluorescently labelled secondary antibodies, the slides were analyzed with a confocal microscope.

**Supplementary materials**

**
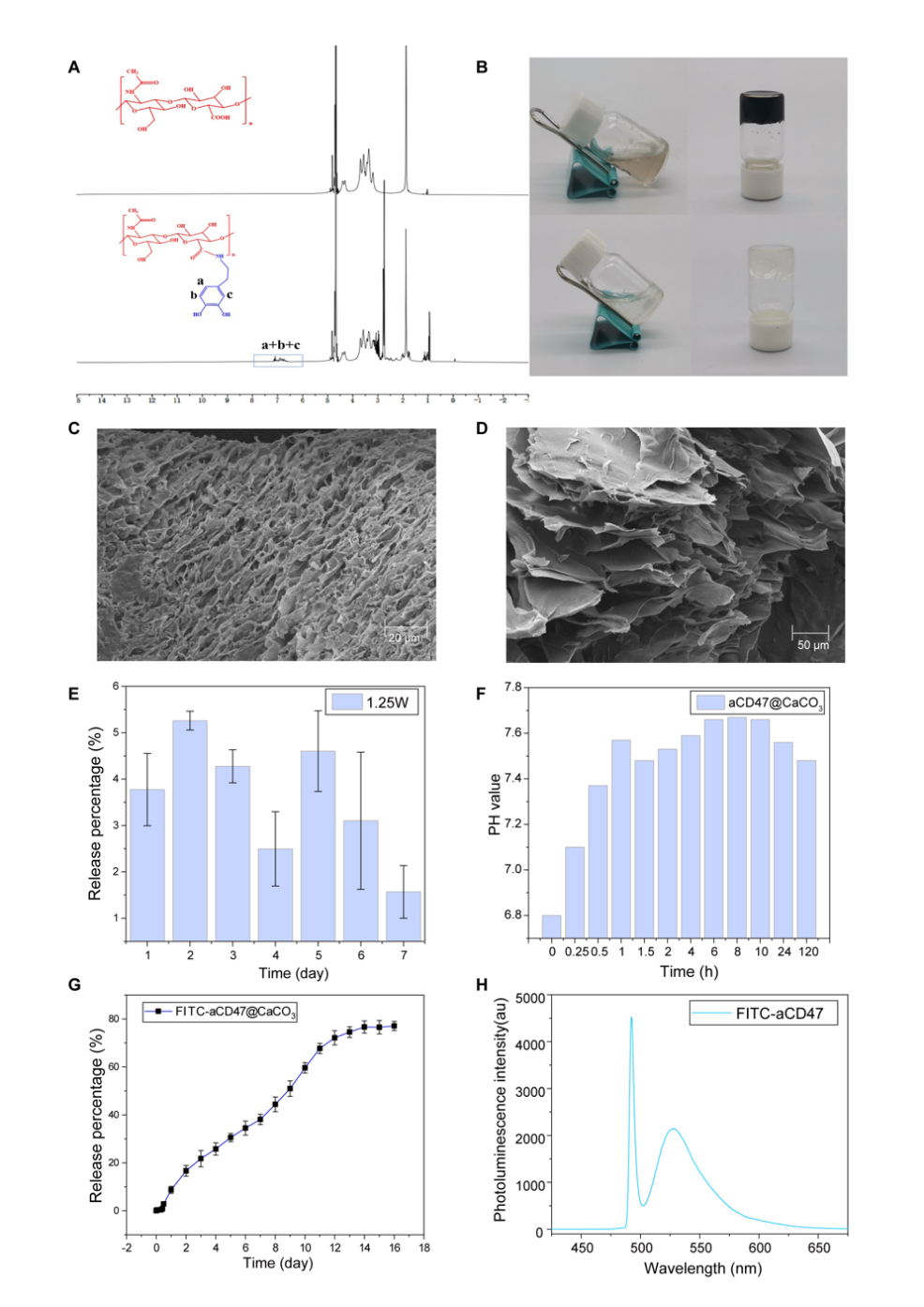
**

**Fig. S1 Synthesis and characterization of the nanocomposite hydrogel.**

(**A**) Nuclear magnetic resonance spectroscopy (^1^H NMR) spectra of HA and HA-DOPA. (**B**) Gelation process of HA-DOPA gel (top) and calcium alginate gel (bottom). (**C**) SEM images of APHP photothermal-photodynamic hydrogel. Scale bar, 20 μm. (**D**) SEM images of CCCA immunomodulatory hydrogel. Scale bar, 50 μm. (**E**) Daily cumulative release profile of δ-ALA from APHP-CCCA hydrogel irradiated by 808 nm laser at 1.25 W for 15 min daily over 1 week. (**F**) The pH changes of supernatant of aCD47@CaCO_3_ immunomodulatory hydrogel in an in vitro simulated tumor microenvironment (PBS, initial pH = 6.8). (**G**) Cumulative release kinetics of FITC-labeled aCD47@CaCO_3_ from APHP-CCCA hydrogel in an in vitro simulated tumor microenvironment (PBS, initial pH 6.8). (**H**) Fluorescence emission spectra of FITC-labeled aCD47. Data are presented as the mean ± SD; *n* = 3 independent experiments.


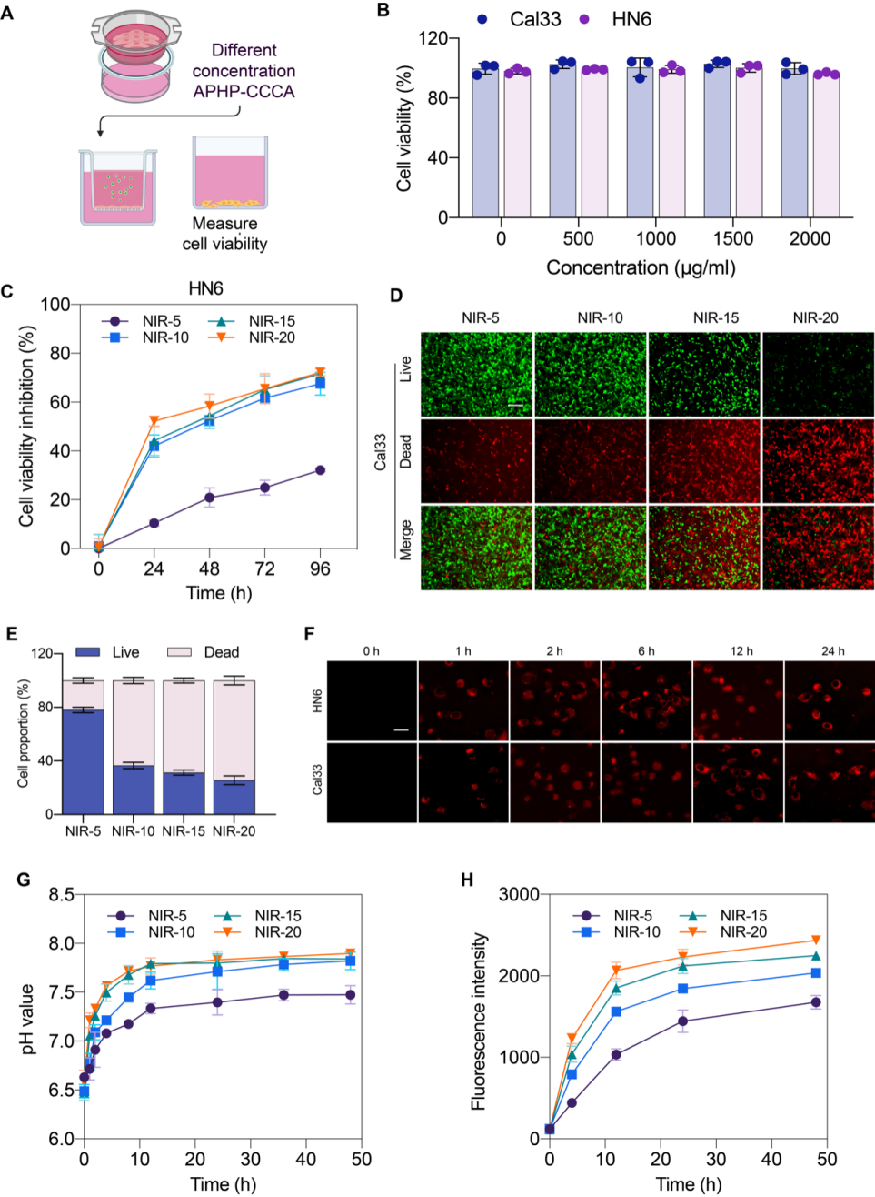


**Fig. S2 Phototherapeutic effects and light-controlled drug release of APHP-CCCA.**

(**A**) Schematic illustration of in vitro cytotoxicity assay of APHP-CCCA on OSCC cell viability. (**B**) In vitro cytotoxicity of APHP-CCCA at different concentrations on OSCC cell lines (Cal33 and HN6) after 72 h incubation without NIR irradiation. (**C**) Time-dependent photothermal therapy-induced cell viability inhibition in HN6 cells treated with 100 μg·mL^-1^ APHP-CCCA upon 808 nm laser irradiation (1.25 W·cm^-2^) for different durations (5, 10, 15, and 20 min; NIR-5, NIR-10, NIR-15 and NIR-20). (**D**) Representative images of Live/Dead staining of APHP-CCCA treated OSCC cells after 808 nm laser irradiation for different durations. Scale bar, 50 μm. (**E**) Quantitative analysis of cell viability based on Live/Dead staining. (**F**) Representative images of intracellular PpIX fluorescence in OSCC cells after 808 nm laser irradiation. Scale bar, 10 μm. (**G**) pH values of OSCC cell supernatant after 808 nm laser irradiation for different durations. (**H**) Cumulative release profiles of FITC-labeled aCD47 from APHP-CCCA hydrogel after 808 nm laser irradiation for different durations. Data are presented as the mean ± SD; *n* = 3 independent experiments. *P* values were determined by two-way ANOVA, Tukey’s multiple-comparison test (ns, not significant; ^*^*P* < 0.05; ^**^*P* < 0.01; ^***^*P* < 0.001).


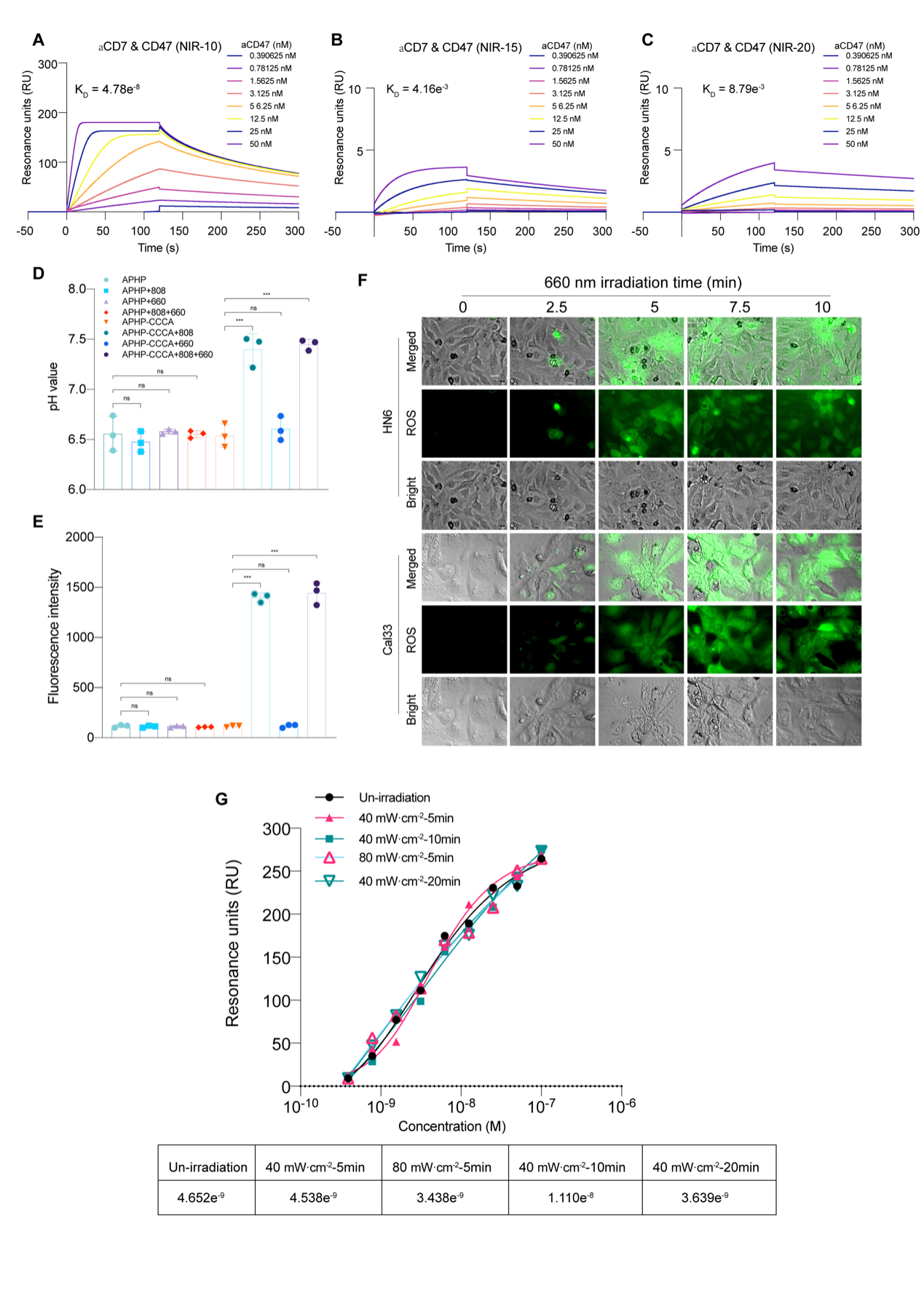


**Fig. S3 Light-controlled aCD47 and δ-ALA release from APHP-CCCA.**

1. **C**) Dose-response curves from SPR measurements showing binding of aCD47 released from APHP-CCCA upon 808 nm laser irradiation at 1.25 W·cm^-2^ for 10 min (**A**), 15 min (**B**) and 20 min (**C**) to immobilized CD47 protein. (**D**) pH values of cell culture supernatants from Cal33 cells treated with APHP or APHP-CCCA and irradiated with 808 nm laser at 1.25 W·cm^-2^ for 5 min and/or 660 nm laser at 40 mW·cm^-2^ for 20 min followed by 24 h incubation. (**E**) Quantitative analysis of FITC-labeled aCD47 release in cell culture supernatants from Cal33 cells treated with APHP or APHP-CCCA and irradiated with 808 nm laser at 1.25 W·cm^-2^ for 5 min and/or 660 nm laser at 40 mW·cm^-2^ for 20 min followed by 24 h incubation. (**F**) ROS levels in Cal33 and HN6 cells visualized by fluorescence microscopy after treatment with 660 nm laser at 40 mW·cm^-2^ for different durations (0, 2.5, 5, 7.5, and 10 min); lower panel: bright field; middle panel: green fluorescence indicating ROS; upper panel: overlay of bright field and green fluorescence images. Scale bar, 10 μm. (**G**) SPR sensorgrams showing binding of aCD47 released from APHP-CCCA hydrogel after 660 nm laser irradiation with 40 or 80 mW·cm^-2^ for 5, 10 or 20 min to immobilized CD47 protein. The K_D_ values are shown in the table below. Data are presented as the mean ± SD; *n* = 3 independent experiments. *P* values were determined by two-way ANOVA, Tukey’s multiple-comparison test (ns, not significant; ^*^*P* < 0.05; ^**^*P* < 0.01; ^***^*P* < 0.001).

**
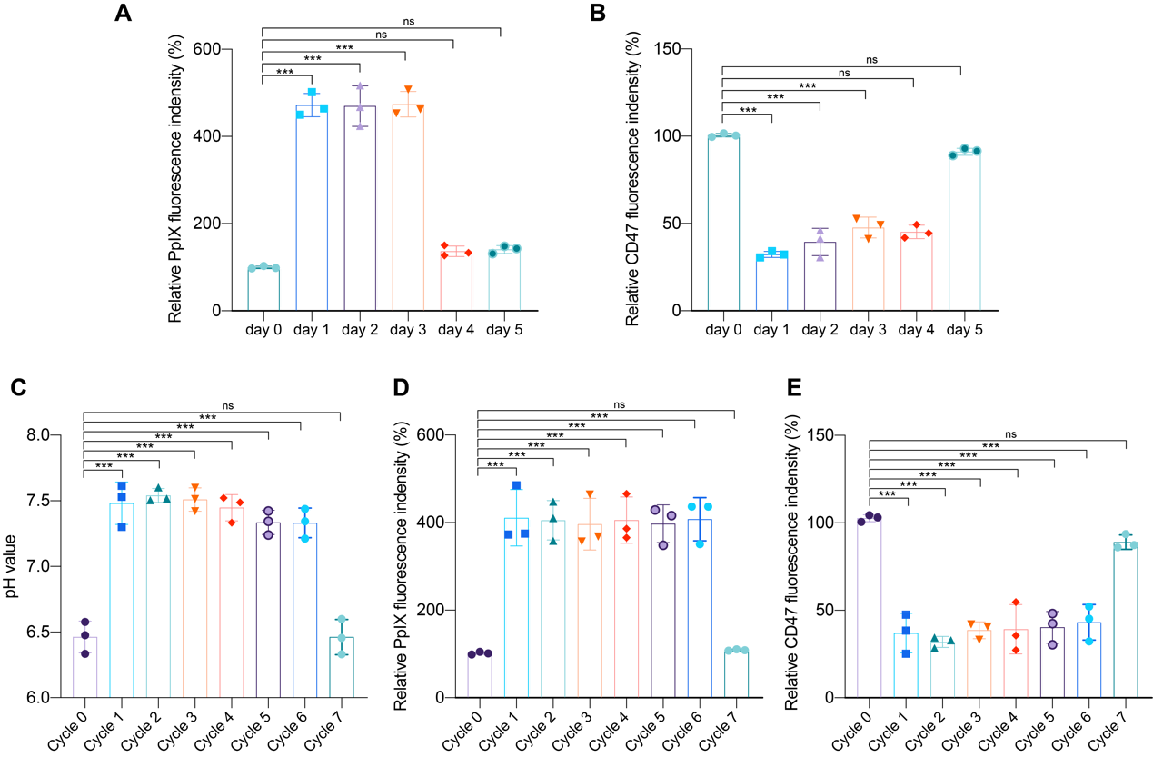
**

**Fig. S4 Detection of drug release and long-term tumor retention of APHP-CCCA in residual tumor tissue.**

(**A**) Quantitative analysis of intracellular PpIX levels in residual tumor tissue on each day for 5 days following surgery after 5 min of 808 nm plus 5 min of 660 nm laser irradiation. (**B**) Quantitative analysis of CD47 expression in residual tumor tissue on each day for 5 days following surgery after 5 min of 808 nm plus 5 min of 660 nm laser irradiation. (**C**) Intratumoral pH values after each irradiation cycle for a total of 7 cycles following surgery. (**D**) Quantitative analysis of intracellular PpIX levels in residual tumor tissue after each irradiation cycle for a total of 7 cycles following surgery. (**E**) Quantitative analysis of CD47 expression in residual tumor tissue after each irradiation cycle for a total of 7 cycles following surgery. Data are presented as the mean ± SD; *n* = 3 independent experiments. *P* values were determined by two-way ANOVA, Tukey’s multiple-comparison test (ns, not significant; ^*^*P* < 0.05; ^**^*P* < 0.01; ^***^*P* < 0.001).

**
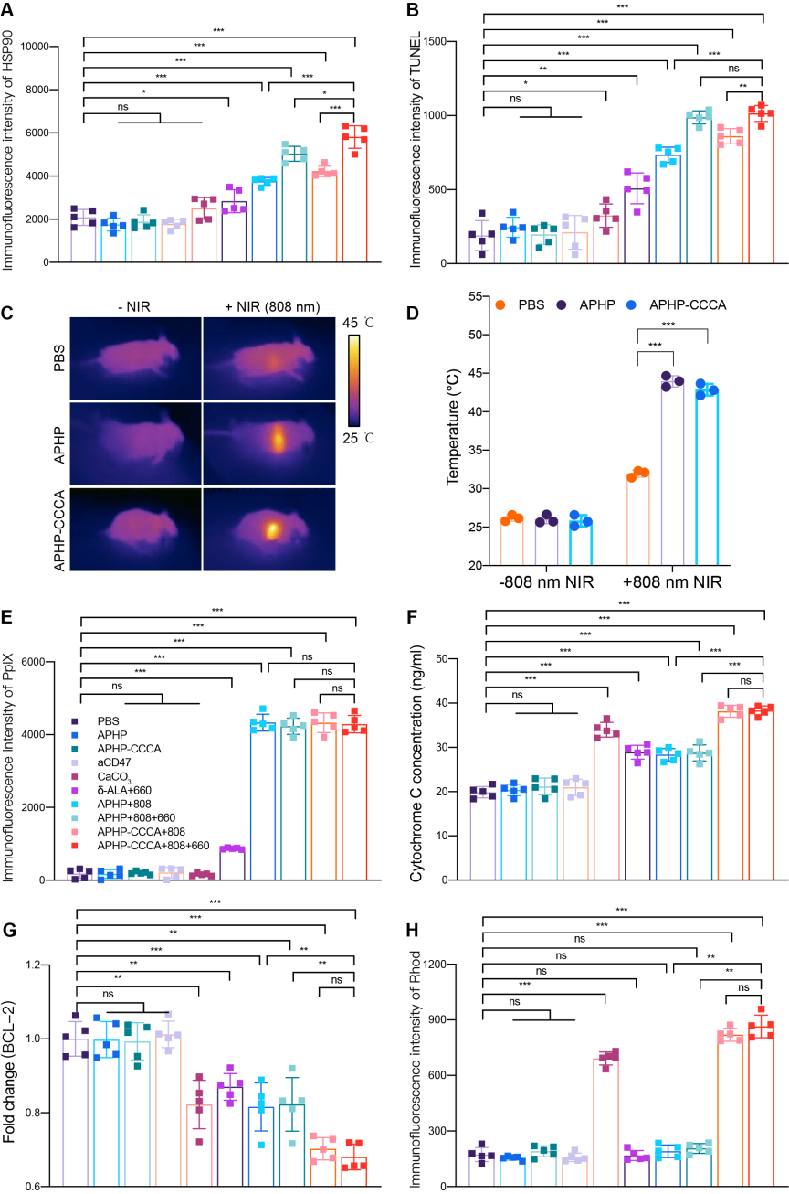
**

**Fig. S5 In vivo tumor recurrence suppression efficacy of APHP-CCCA hydrogel implantation postsurgery.**

(**A**) Quantitative analysis of HSP90 immunofluorescence staining of residual tumor tissues obtained from mice in each group on the third day after surgery. (**B**) Quantitative analysis of TUNEL staining of residual tumor tissues obtained from mice in each group on the third day after surgery. (**C**) Thermal images of SCC7 tumor-bearing mice following implantation of PBS, APHP, or APHP-CCCA with or without 808 nm laser irradiation at a power density of 1.25 W·cm^-2^ for 5 min. (**D**) Quantitative analysis of temperature of SCC7 tumor-bearing mice following implantation of PBS, APHP, or APHP-CCCA with or without 808 nm laser irradiation at a power density of 1.25 W·cm^-2^ for 5 min. (**E**) Quantitative analysis of intracellular PpIX in residual tumor tissues obtained from mice in each group on the third day after surgery. (**F**) Quantitative analysis of cytochrome C concentration from ELISA kits in residual tumor tissues obtained from mice in each group on the third day after surgery. (**G**) RT-qPCR detection of Bcl-2 expression in residual tumor tissues obtained from mice in each group on the third day after surgery. (**H**) Quantitative analysis of fluorescence images showing calcium influx in live Cal33 cells from each group. Data are presented as the mean ± SD; *n* = 3 independent experiments. *P* values were determined by two-way ANOVA, Tukey’s multiple-comparison test (ns, not significant; ^*^*P* < 0.05; ^**^*P* < 0.01; ^***^*P* < 0.001).


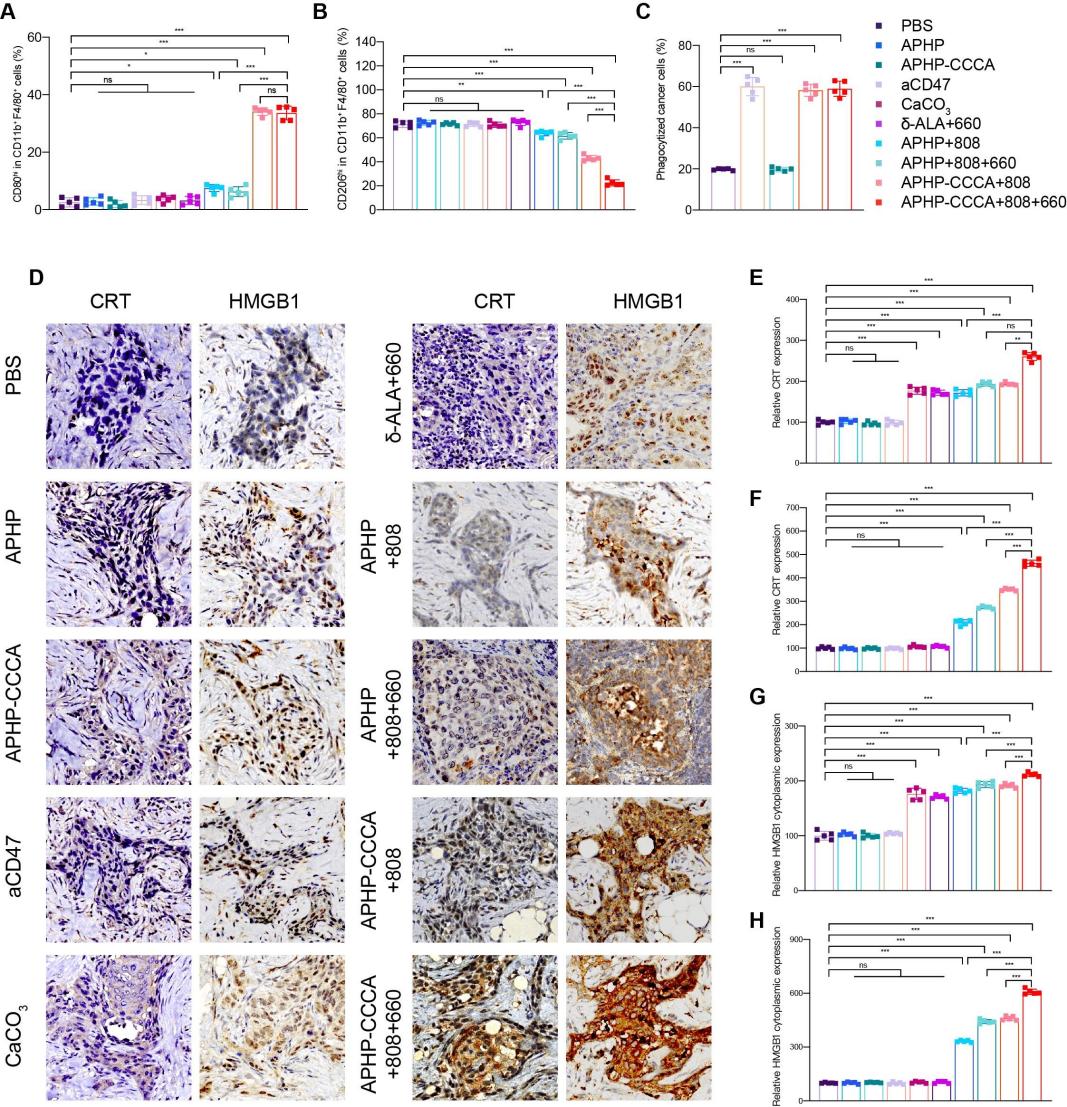


**Fig. S6 In vivo immunomodulatory effects of APHP-CCCA hydrogel implantation postsurgery in an OSCC model.**

(**A-B**) Relative quantifications of the proportions of pro-inflammatory M1-like TAMs (**A**) and anti-inflammatory M2-like TAMs (**B**) as percentages of total F4/80^+^CD11b^+^ TAM populations in residual OSCC tumor tissues obtained from mice in each experimental group on day 15 after implantation surgery. (**C**) Relative quantification of the phagocytic uptake of HN6 cells by RAW264.7 macrophages in vitro. (**D**) Immunohistochemical staining detecting CRT expression and nuclear versus cytoplasmic localization of the damage-associated molecular pattern protein HMGB1 in residual OSCC tumor tissues obtained from mice in each experimental group on day 15 after implantation. Scale bar, 50 μm. (**E-F**) Relative quantification of immunohistochemical staining detecting CRT expression in residual OSCC tumor tissues obtained from mice in each experimental group on day 3 (**E**) and day 15 (**F**) after implantation. (**G-H**) Relative quantification of immunohistochemical staining detecting HMGB1 expression in residual OSCC tumor tissues obtained from mice in each experimental group on day 3 (**G**) and day 15 (**H**) after hydrogel implantation. Data are presented as the mean ± SD; *n* = 3 independent experiments. *P* values were determined by two-way ANOVA, Tukey’s multiple-comparison test (ns, not significant; ^*^*P* < 0.05; ^**^*P* < 0.01; ^***^*P* < 0.001).


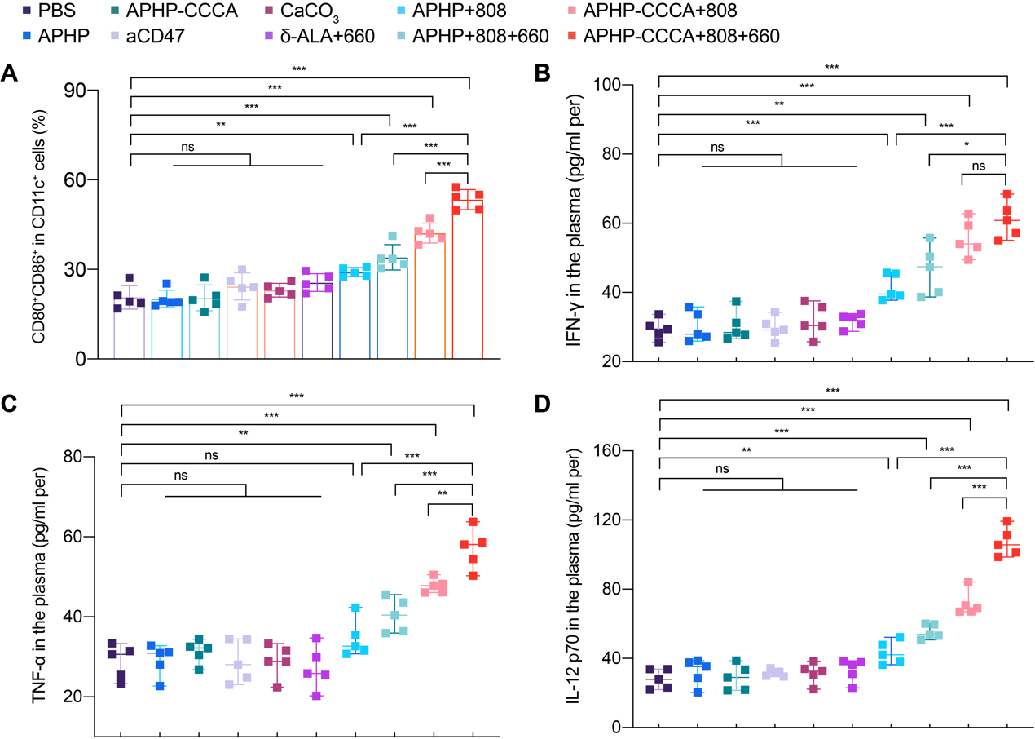


**Fig. S7 Antitumor immune response induced by implantation of APHP-CCCA hydrogel postsurgery.**

(**A**) Relative quantification by flow cytometry of the proportion of mature DCs (CD11c^+^CD80^+^CD86^+^) in the tumor-draining lymph nodes of mice in each experimental group on day 15 post-implantation. (**B-D**) Relative quantifications of serum levels of typical immune-related cytokines, including IFN-γ (**B**), TNF-α (**C**), and IL-2 (**D**), measured by ELISA. Data are presented as the mean ± SD; *n* = 3 independent experiments. *P* values were determined by two-way ANOVA, Tukey’s multiple-comparison test (ns, not significant; ^*^*P* < 0.05; ^*^**P* < 0.01; ^***^*P* < 0.001).


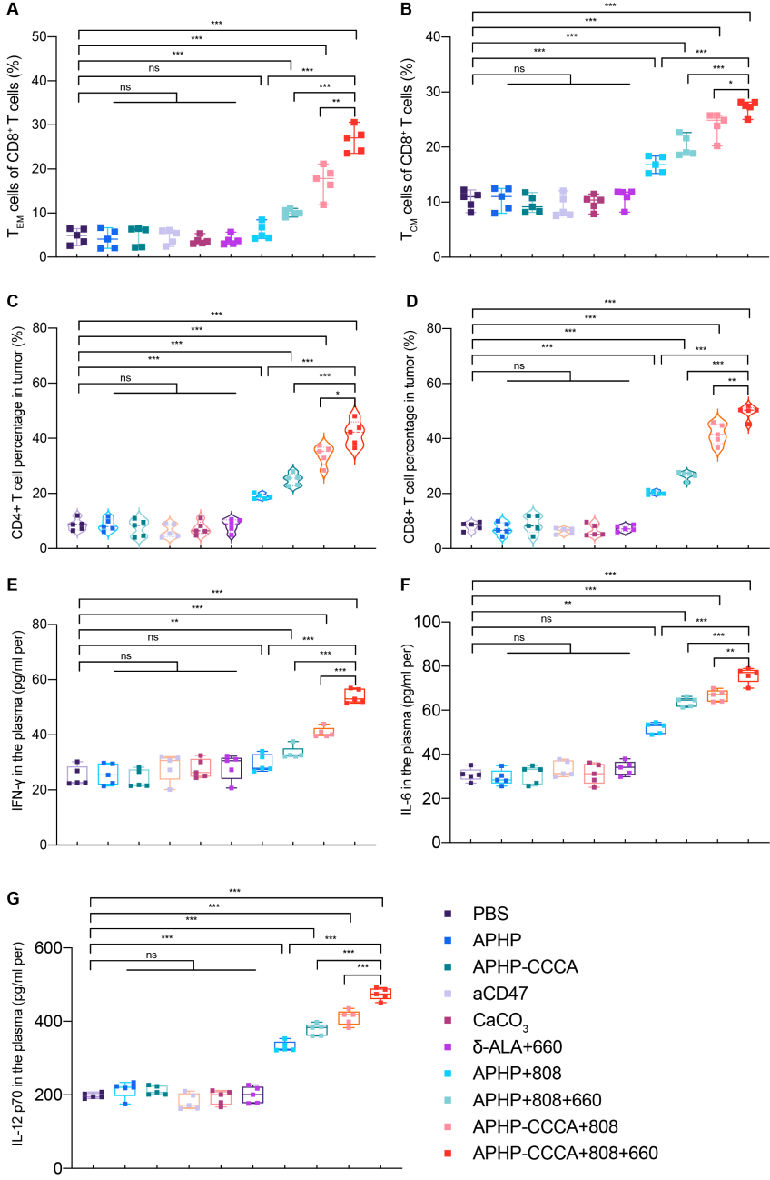


**Fig. S8 Abscopal anti-tumor effects induced by APHP-CCCA hydrogel implantation postsurgery in OSCC tumors**

(**A-B**) Representative quantification of flow cytometry analyses of systemic anti-tumor immune responses in PBMCs isolated from mice in each experimental group. T_EM_ (CD62L^−^CD44^+^CD8^+^) (**A**) and T_CM_ (CD62L^+^CD44^+^CD8^+^) (**B**) were quantified at the day 30 after hydrogel implantation. (**C-D**) Representative quantification of immunohistochemical staining detecting CD4^+^ (**C**) and CD8^+^ (**D**) T cell infiltration within distant secondary OSCC tumors obtained on day 30 post-implantation. (**E-G**) Quantitative ELISA analysis of IFN-γ (**E**), IL-6 (**F**), and IL-12 p70 (**G**) in serum obtained from mice in each group on day 30 post-implantation. Data are presented as the mean ± SD; *n* = 3 independent experiments. *P* values were determined by two-way ANOVA, Tukey’s multiple-comparison test (ns, not significant; ^*^*P* < 0.05; ^**^*P* < 0.01; ^***^*P* < 0.001).


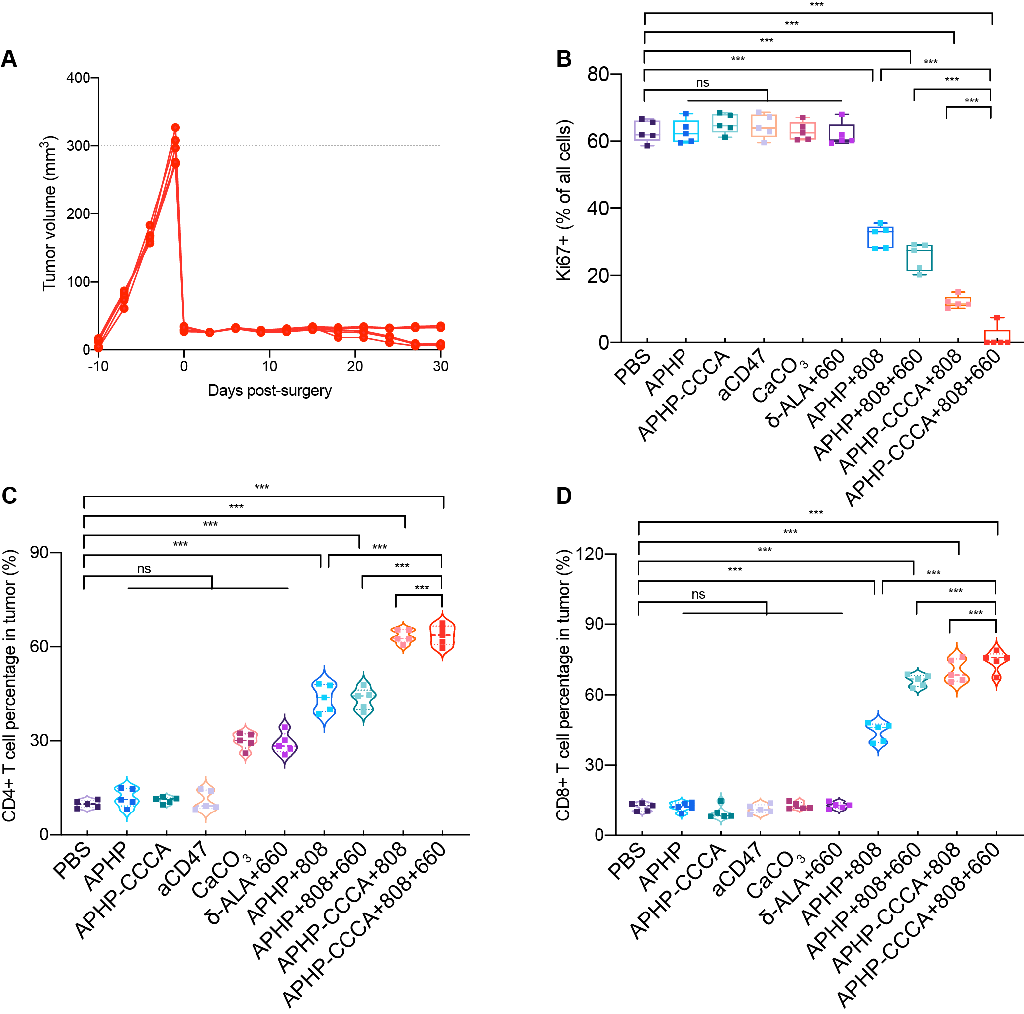


**Fig. S9 Abscopal effect of APHP-CCCA in preventing orthotopic OSCC tongue tumors and lung metastasis.**

1. Individual growth curves of recurrent flank tumors in mice treated with "APHP-CCCA+808+660" over 30 days following tumor resection and hydrogel implantation. (**B**) Relative quantification of immunohistochemical staining detecting Ki67 expression in orthotopic tongue tumors from each experimental group at day 30 post-surgery. (**C-D**) Relative quantification of immunofluorescence staining showing infiltration of CD4^+^ (**C**) and CD8^+^ (**D**) T cells within orthotopic tongue tumors at day 30 postsurgery and hydrogel implantation. Data are presented as the mean ± SD; *n* = 3 independent experiments. *P* values were determined by two-way ANOVA, Tukey’s multiple-comparison test (ns, not significant; ^*^*P* < 0.05; ^**^*P* < 0.01; ^***^*P* < 0.001).


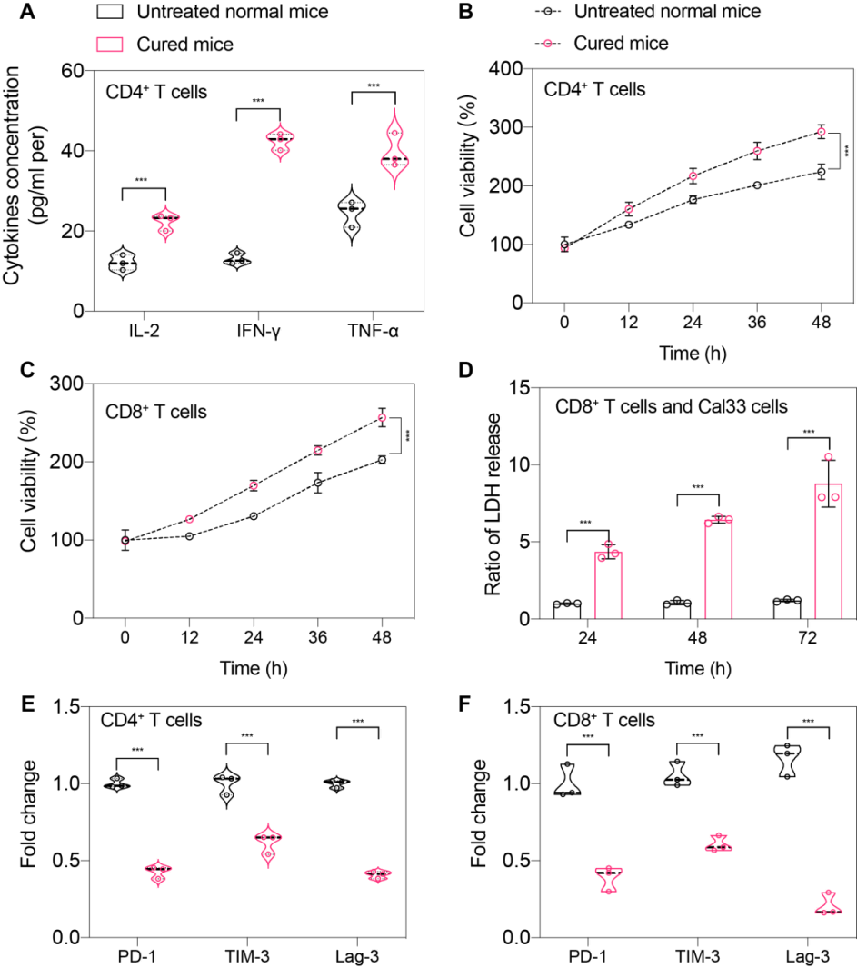


**Fig. S10. Impact of APHP-CCCA on T cell functionality.**

1. Cytokine analysis of inflammatory mediators IFN-γ, TNF-α, and IL-2 from isolated CDK4^+^ T cells with magnetic beads from untreated normal mice and mice cured with the "APHP-CCCA+808+660" treatment. (**B-C**) Cell viability of CD4^+^ (**B**) and CD8^+^ (**C**) T cells from untreated normal mice and mice cured with the "APHP-CCCA+808+660" treatment, stimulated with the activating agent ConA. (**D**) LDH release assays measuring lactate dehydrogenase enzyme activity released from damaged tumor cells cocultured with "APHP-CCCA+808+660" or untreated CD8^+^ T cells with Cal33 cells. (**E-F**) Expression of exhaustion marker genes (PD-1, TIM-3, LAG-3) on CD4^+^ (**E**) and CD8^+^ (**F**) T cells under different treatment conditions using RT-qPCR. Data are presented as the mean ± SD; *n* = 3 independent experiments. *P* values were determined by two-way ANOVA, Tukey’s multiple-comparison test (ns, not significant; ^*^*P* < 0.05; ^**^*P* < 0.01; ^***^*P* < 0.001).


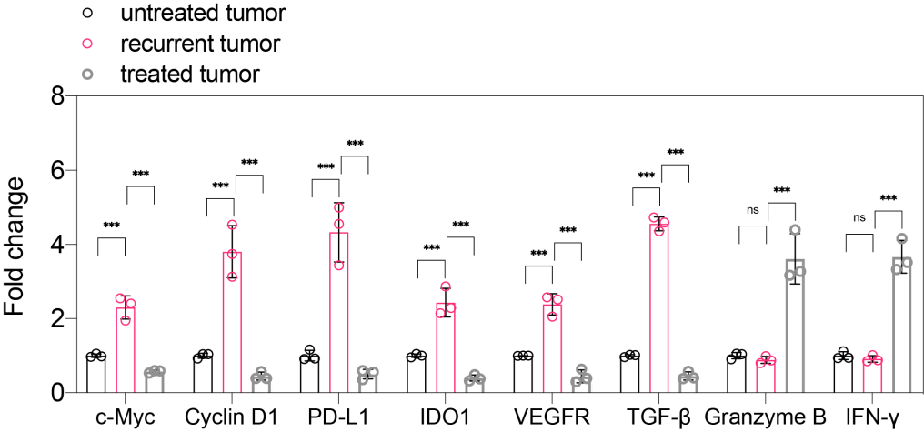


**Fig. S11. Gene expression in untreated tumors, recurrent tumors postsurgery, and tumors treated with hydrogel vaccine.** Data are presented as the mean ± SD; *n* = 3 independent experiments. *P* values were determined by two-way ANOVA, Tukey’s multiple-comparison test (ns, not significant; ^*^*P* < 0.05; ^**^*P* < 0.01; ^***^*P* < 0.001).


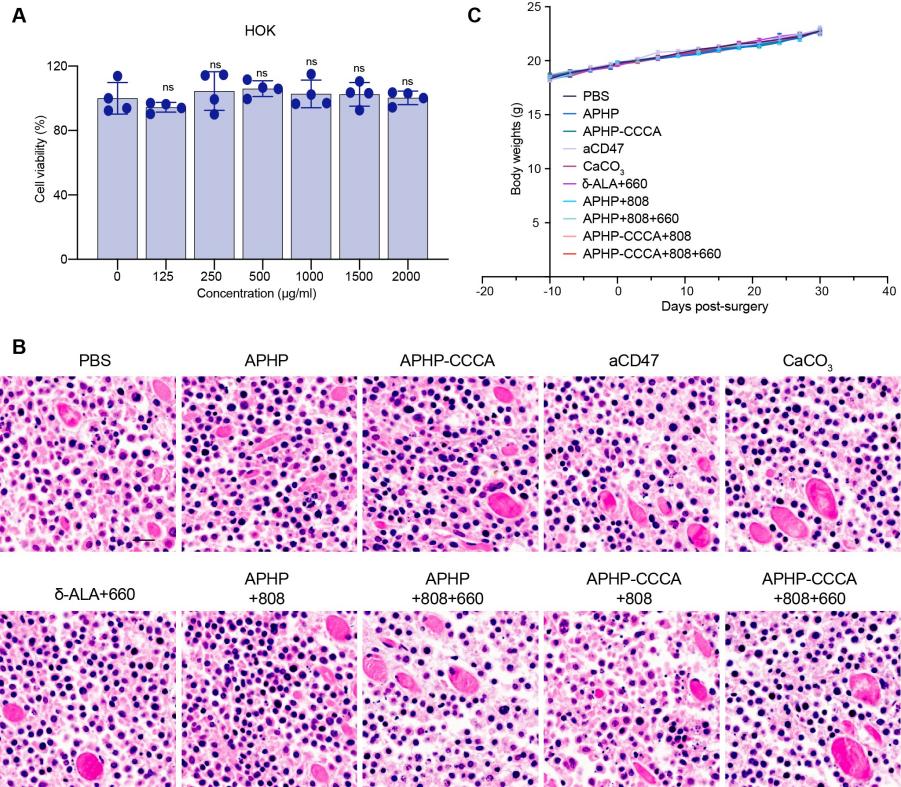


**Fig. S12 Biosafety assessment of in situ vaccination with APHP-CCCA.**

1. Cell viability of HOK cells treated with varying concentrations of APHP-CCCA hydrogel leachate solution in the absence of NIR irradiation. (**B**) H&E staining of normal tissues surrounding the hydrogel implantation site at 4 week post-treatment. Scale bar, 10 μm. (**C**) Body weight measurements of mice in each experimental group over 30 days following surgery and hydrogel implantation. Data are presented as the mean ± SD; *n* = 3 independent experiments. *P* values were determined by two-way ANOVA, Tukey’s multiple-comparison test (ns, not significant; ^*^*P* < 0.05; ^**^*P* < 0.01; ^***^*P* < 0.001).


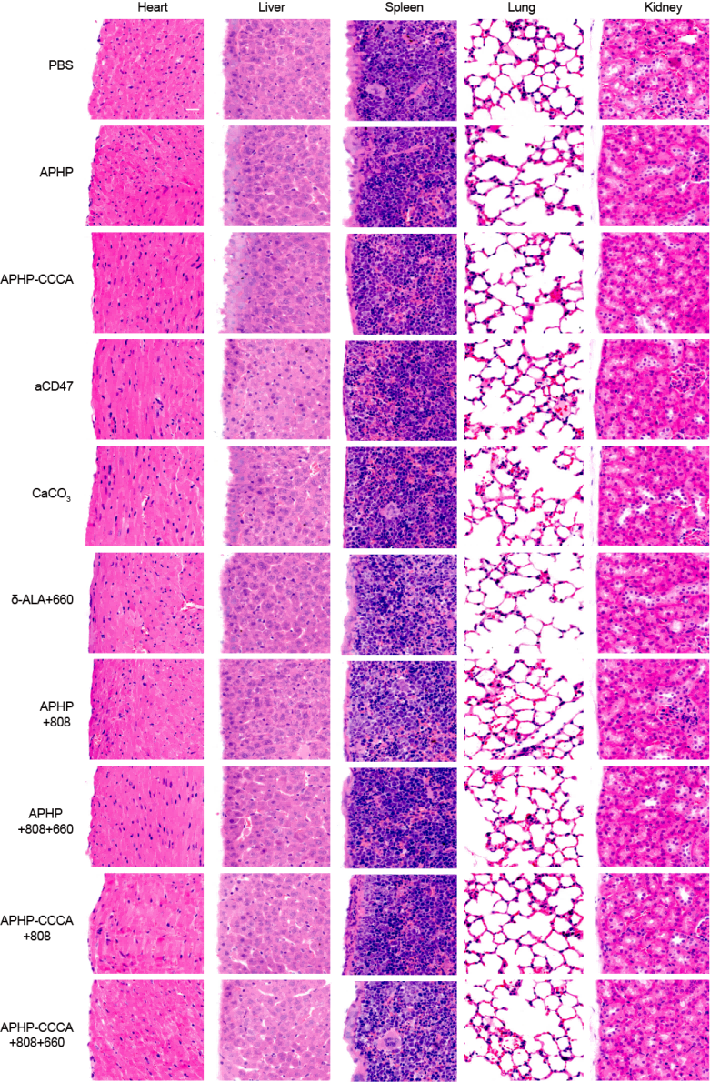


**Fig. S13 H&E images of main organs of mice in each experimental group over 30 days following surgery and hydrogel implantation. Scale bar, 20 μm.**

**
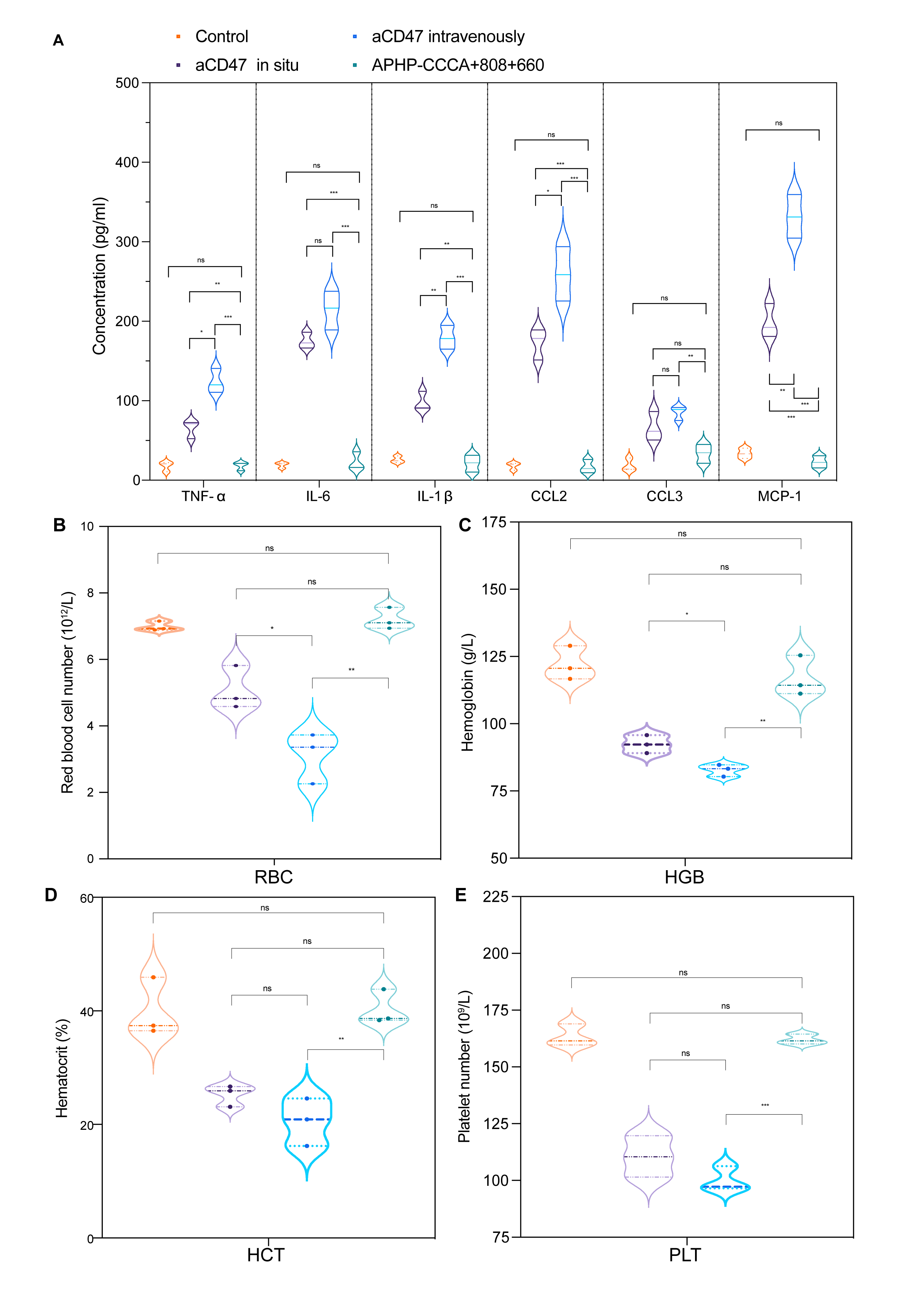
**

**Fig. S14 Hematological safety analysis following APHP-CCCA hydrogel implantation.**

(**A**) Quantitative ELISA analysis of cytokine and chemokine levels in serum obtained from mice in each experimental group at 6 h post-implantation. (**B-E**) Quantitative analysis of complete blood count parameters including red blood cell number (RBC), hemoglobin (HGB), hematocrit (HCT), and platelet number (PLT) in mice from each experimental group at 6 h after hydrogel implantation. Data are presented as the mean ± SD; *n* = 3 independent experiments. *P* values were determined by two-way ANOVA, Tukey’s multiple-comparison test (ns, not significant; ^*^*P* < 0.05; ^**^*P* < 0.01; ^***^*P* < 0.001).


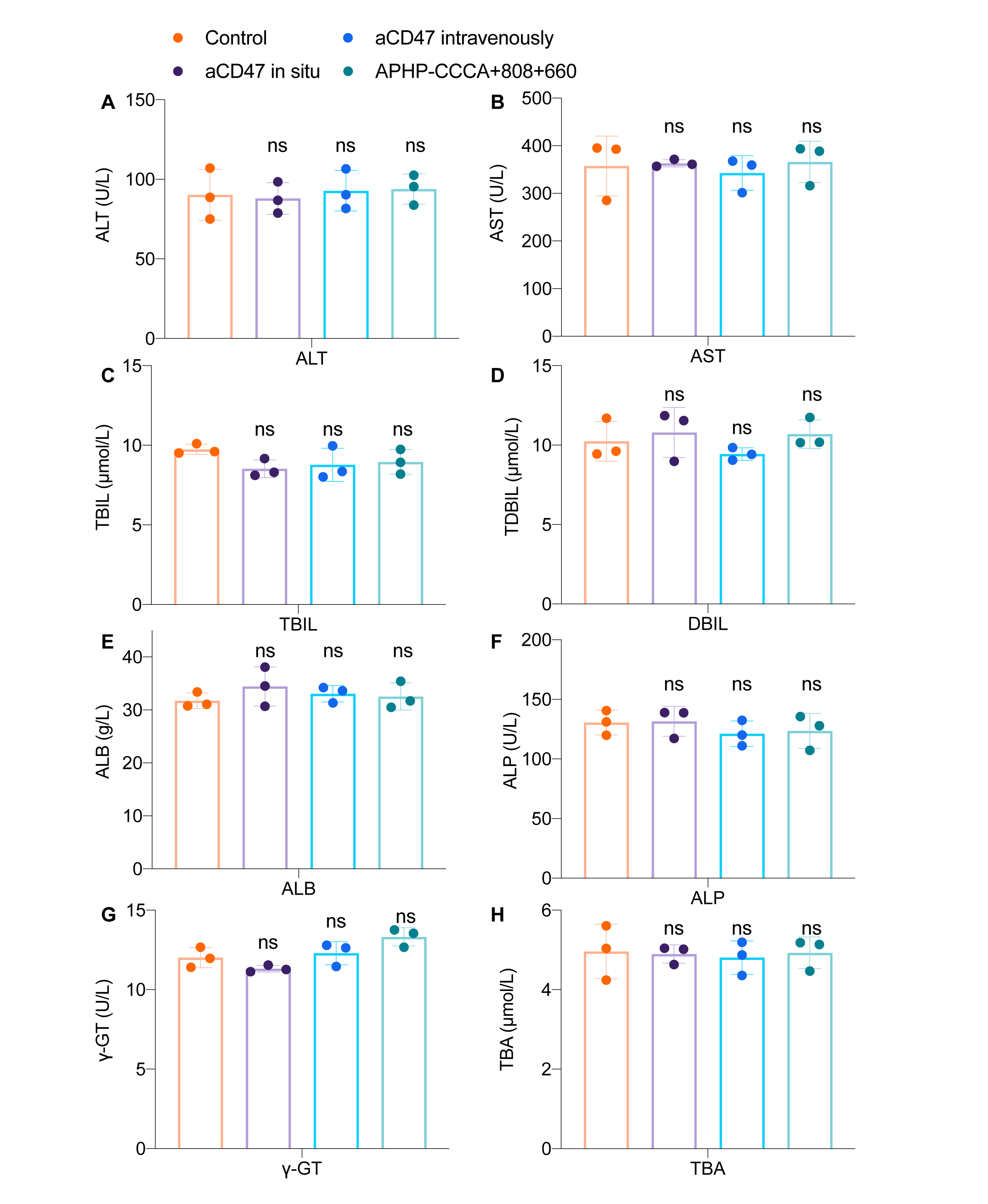


**Fig. S15 Serum biochemistry analysis following APHP-CCCA hydrogel implantation.**

Quantitative serum biochemistry assays were performed on blood samples collected at 6 h after implantation of APHP-CCCA hydrogel or control treatments. The following hepatic and renal function markers were analyzed: Alanine aminotransferase (ALT); Aspartate aminotransferase (AST); Total bilirubin (TBIL); Direct bilirubin (DBIL); Albumin (ALB); Alkaline phosphatase (ALP); Gamma-glutamyl transferase (γ-GT); and Total bile acid (TBA). Data are presented as the mean ± SD; *n* = 3 independent experiments. *P* values were determined by two-way ANOVA, Tukey’s multiple-comparison test (ns, not significant; ^*^*P* < 0.05; ^**^*P* < 0.01; ^***^*P* < 0.001).
